# Structural and biochemical characteristics of two *Staphylococcus epidermidis* RNase J paralogs RNase J1 and RNase J2

**DOI:** 10.1074/jbc.RA120.014876

**Published:** 2021-01-13

**Authors:** Rishi Raj, Savitha Nadig, Twinkal Patel, Balasubramanian Gopal

**Affiliations:** Molecular Biophysics Unit, Indian Institute of Science, Bangalore, India

**Keywords:** RNA recycling, RNase J paralogs, metalloribonucleases, metal cofactors, RNA degradosome, exoribonuclease II (Rnase II), metalloenzyme, oligomerization, protein-protein interaction, metal ion–protein interaction

## Abstract

RNase J enzymes are metallohydrolases that are involved in RNA maturation and RNA recycling, govern gene expression in bacteria, and catalyze both exonuclease and endonuclease activity. The catalytic activity of RNase J is regulated by multiple mechanisms which include oligomerization, conformational changes to aid substrate recognition, and the metal cofactor at the active site. However, little is known of how RNase J paralogs differ in expression and activity. Here we describe structural and biochemical features of two *Staphylococcus epidermidis* RNase J paralogs, RNase J1 and RNase J2. RNase J1 is a homodimer with exonuclease activity aided by two metal cofactors at the active site. RNase J2, on the other hand, has endonuclease activity and one metal ion at the active site and is predominantly a monomer. We note that the expression levels of these enzymes vary across *Staphylococcal* strains. Together, these observations suggest that multiple interacting RNase J paralogs could provide a strategy for functional improvisation utilizing differences in intracellular concentration, quaternary structure, and distinct active site architecture despite overall structural similarity.

RNA modification and degrading enzymes regulate diverse cellular processes in bacteria (1). RNA maturation and recycling mediated by exo- and endoribonucleases alongside other RNA-binding proteins also influence changes in gene expression. Despite similar functional requirements, these RNA degradation mechanisms vary substantially across bacteria. This diversity is best illustrated by the differences in the composition of the multienzyme RNA degradosome complex (2). In *Escherichia coli*, for example, RNase E, polynucleotide phosphorylase (PNPase), RhlB, and enolase are degradosome components with RNase E forming the scaffold for this multienzyme assembly (2). On the other hand, Gram-positive bacteria such as *Bacillus subtilis* or *Staphylococcus aureus* lack RNase E. In these Firmicutes, the functional homolog is now generally accepted to be RNase Y, although RNase J was proposed initially (3, 4, 5).

Most Firmicutes have multiple RNase J paralogs (3, 4). Both RNase J paralogs in *B. subtilis* and *S. aureus*, referred to as RNase J1 and RNase J2, along with RNase Y have been suggested to be components of the RNA degradosome (5, 6). RNase J paralogs are essential for the survival of some but not all Gram-positive bacteria. For example, whereas both paralogs RNase J1 and RNase J2 are essential in *Streptococcus pyogenes* (7, 8), RNase J1 has been shown not to be essential in *B. subtilis* (9). This finding was consistent with the observation that depletion of RNase J1 and RNase J2 in *B. subtilis* only slightly increased the *t*_1/2_ of intracellular mRNA (from 2.6 to 3.6 min) (4). In other studies involving transcriptome and proteome analysis, both single (*rnj1*) and double (*rnj1* and *rnj2*) depletion mutants in *B. subtilis* strains were found to have substantial differences in the expression levels of multiple genes, suggesting that RNase J1 plays an extensive role in modulating gene expression (10, 11). Similar observations were made in the case of the *S. aureus* RNase J paralogs wherein *S. aureus* RNase J1 was first thought to be essential (12). In *S. aureus*, deletion of genes coding for the two RNase J paralogs yielded a strain that was only viable at 37 °C and not at temperatures either above or below (13). RNase J paralogs have also been shown to influence virulence. For example, *Enterococcus faecalis* RNase J2 is a regulator of the *ebp* operon (encoding endocarditis and biofilm-associated pili), and the Δ*rnjB* strain (deletion of RNase J2) was attenuated in its ability to form biofilms (14). In studies to evaluate the impact of this enzyme on bacterial virulence in a sublethally challenged mouse model, the Δ*rnjB* mutant strain was seen to be less virulent than the WT. Studies in *Streptococcus mutans* also show that *rnj1* and *rnj2* deletion mutants were viable but experienced severe defects in growth, morphology, acid tolerance, natural competence, and biofilm formation, with these features being more severe in RNase J2 deletion mutants (15). These findings thus suggest that RNase J paralogs play distinct roles in these bacteria.

The catalytic activity of RNase J paralogs differs across bacteria (4). In *B. subtilis*, both RNase J1 and RNase J2 have 5′ exoribonuclease and endoribonuclease activity, with the latter being less catalytically active (16). *B. subtilis* RNase J1 was shown to prefer 5′ mono- over 5′ triphosphorylated RNA for exoribonuclease activity (17, 18). *S. aureus* RNase J1, on the other hand, was shown to recognize and cleave both substrates (5′ mono- and 5′ triphosphorylated RNA) in a similar manner (19). Surface plasmon resonance and analytical size exclusion chromatography analysis suggested that *B. subtilis* RNase J1 and RNase J2 interact and form a heteromeric complex (16, 20). However, the stability of the *B. subtilis* RNase J1/RNase J2 complex was seen to be sensitive to the time taken for protein preparation, concentration of the two enzymes, and buffer conditions (16).

Structures of RNase J enzymes from *Thermus thermophilus*, *B. subtilis*, *Streptomyces coelicolor*, *Dienococcus radiodurans*, and *Methanolobus psychrophilus* reveal multiple conserved features which include the RNA substrate recruitment interface and the catalytic center ensconced between the β-lactamase and the β-CASP domain (17, 20, 21, 22, 23). All reported RNase J enzymes are multimeric, either homodimers or homotetramers. The structure of *D. radiodurans* RNase J bound to uridine monophosphate suggested that a manganese ion is crucial for the dimeric assembly (21). On the other hand, *S. coelicolor*, *B. subtilis*, *T. thermophilus*, and *M. psychrophilus* RNase J enzymes dimerize independent of a metal nucleant (17, 20, 21, 22). All characterized RNase J homologs, however, rely on a metal cofactor for catalytic activity. For example, in *S. coelicolor*, *D. radiodurans*, and *M. psychrophilus*, RNase J has two zinc ions at the active site. This observation suggested that a catalytic mechanism involving two metal ions could be a general feature facilitating the generation of hydroxyl ions for nucleophilic attack on the phosphodiester bond (17, 22, 23).

The dual (endo- and exoRNase) activity of RNase J is also influenced by the quaternary structure of this enzyme. An illustration of this feature is *D. radiodurans* RNase J, which is a Mn^2+^-dependent dimer (22). Mutation of any of the residues coordinating the metal ion at the interface led to monomeric units with decreased exoribonuclease activity (22). A surprising finding, however, was that a monomeric (D61A) mutant of *D. radiodurans* RNase J had lower exo- but higher endoribonuclease activity than WT RNase J. A conformational rationale for this observation came from the structure of *B. subtilis* RNase J1 bound to RNA. From this structure, it was evident that dissociation to the monomeric state is essential for endoRNase activity because access to the RNA substrate is blocked by the other subunit in the dimer (21). The quaternary structure, especially in bacterial RNase J homologs, is substantially influenced by the C-terminal polypeptide segment. Removal of the C-terminal polypeptide resulted in a monomeric enzyme with impaired exoribonuclease activity (22). Finally, interactions with the RNA substrate modulate the catalytic function of RNase J. This feature is best illustrated in the case of *S. coelicolor* RNase J wherein mutation of specific RNA-binding residues triggered a switch between exoribonuclease and endoribonuclease activities (17). The known factors that contribute to differences in RNase J function are thus diverse, involving oligomerization, metal ion binding, and/or RNA interactions (17).

Here we describe studies on two RNase J paralogs: RNase J1 and RNase J2 from *Staphylococcus epidermidis*. The focus was to evaluate a structural or biochemical rationale for multiple RNase J paralogs in some Firmicutes, such as *Staphylococci.* In particular, we sought to understand the structural role of RNase J2 that has been ascribed to this paralog in the context of RNA degradation (13). A comparison of biochemical features of the two paralogs reveals that the dimeric RNase J1 enzyme has more exoribonuclease than endoribonuclease activity; the opposite is true for the predominantly monomeric RNase J2. Removal of the C-terminal domain in RNase J1 alters the activity profile; the monomeric truncated RNase J1 enzyme has more endoribonuclease activity than the WT enzyme. Comparing the structures of the two *S. epidermidis* paralogs with other RNase J enzymes revealed that the active site of RNase J2 differs from most other RNase J enzymes characterized thus far. This enzyme has one metal ion (Mn^2+^) bound at the active site. Furthermore, Ca^2+^ is the most preferred metal cofactor for RNase J2 activity *in vitro*. We also note that the expression levels of the two RNase J paralogs vary across *Staphylococcal* strains. Together, these observations suggest that multiple RNase J paralogs provide an evolutionary route for functional optimization within the conserved RNase J scaffold.

## Results

### The two S. epidermidis RNase J paralogs differ in their quaternary arrangement

**Figure 1:**
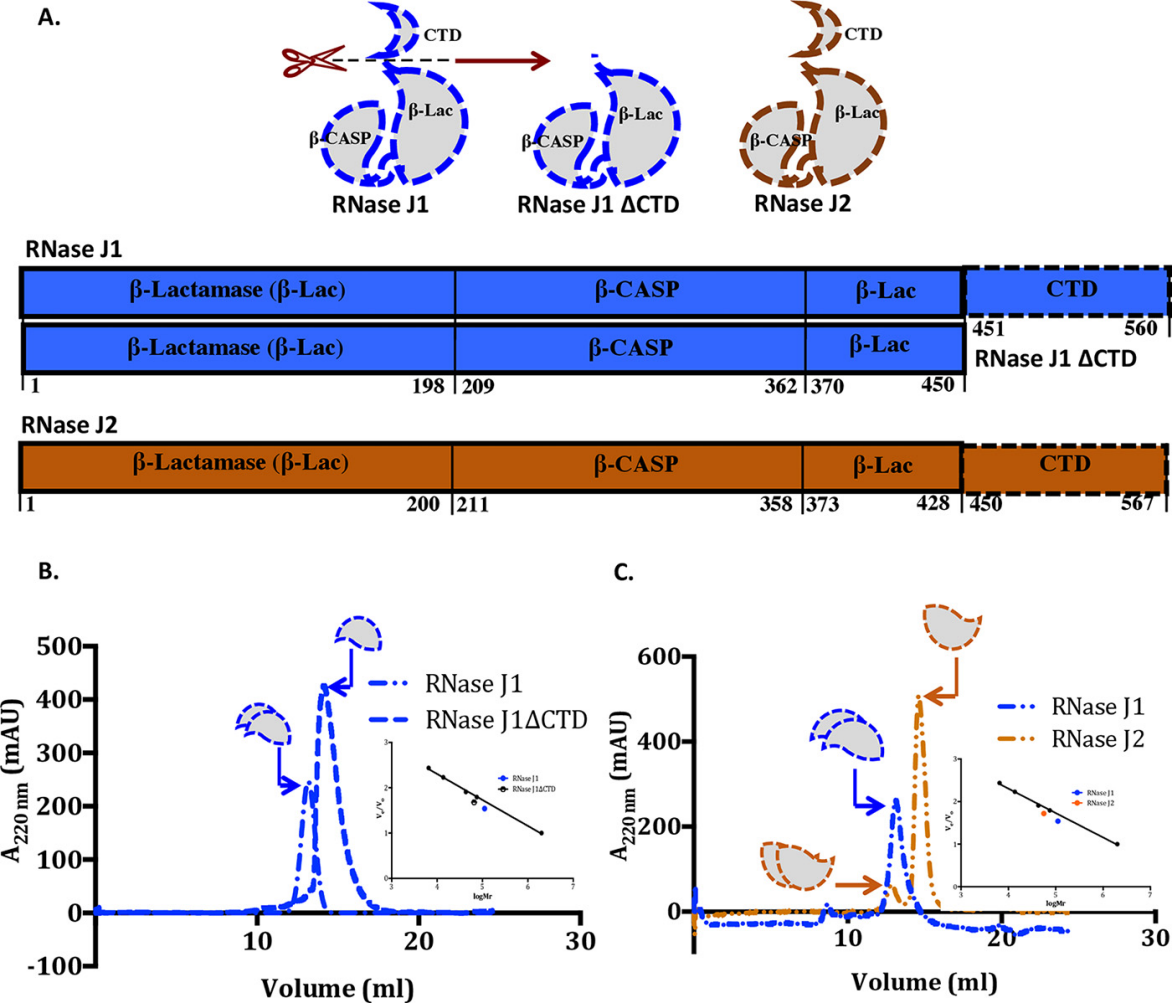
**The quaternary structure governs catalytic activity in RNase J.***A*, domain organization of RNase J paralogs. The N-terminal polypeptide segment contains the catalytic site (located between the β-lactamase and β-CASP domains). The C-terminal domain influences oligomerization and catalysis. *B*, SEC experiments reveal that RNase J1 is a dimer in solution. Removal of the C-terminal domain alters the quaternary association from a homodimer to a monomer. The quaternary structure of this enzyme was also evaluated in a SEC-MALS experiment (Fig. S1). *C*, RNase J2 is predominantly a monomer in solution. This finding is consistent with results from a coexpression and copurification experiment involving RNase J1 and RNase J2. These data (Fig. S2) revealed RNase J2 in stoichiometric excess in the copurified protein sample, thereby suggesting that RNase J2 can exist as a monomer on its own.

The quaternary structure of full-length RNase J1 (62,051 Da) and RNase J2 (62,677 Da) was evaluated by size exclusion chromatography (SEC). We note that RNase J1 is a dimer in solution whereas RNase J2 is seen to be a mostly monomeric enzyme with a minor dimeric species (Fig. 1). Subsequently, SEC–multi angle light scattering (SEC-MALS) experiments were performed on a miniDAWN TREOS instrument. The molecular weight estimates derived from the SEC-MALS data are consistent with the SEC experiments; whereas RNase J1 is a dimer, RNase J2 is monomeric in solution (Fig. S1). In the light of previous reports that the two *B. subtilis* RNase J paralogs interact, recombinant *S. epidermidis* RNase J1 and RNase J2 were coexpressed and purified (Fig. S2). We note that RNase J2 with a poly-histidine tag at the C terminus could pull down RNase J1. It thus appears likely that the two *S. epidermidis* paralogs may interact, as noted earlier in the case of *B. subtilis* RNase J1 and RNase J2. The yield of *S. epidermidis* RNase J2 that could be obtained in this experiment is more than RNase J1, consistent with the observation that RNase J2 can exist as a monomer on its own (Fig. S2). The low-affinity interactions between the two paralogs, however, limit any inferences to be drawn on the interaction stoichiometry or change in quaternary structure, if any. This finding suggests differences with the observations made on the *B. subtilis* RNase J paralogs. Association between the *B. subtilis* RNase J paralogs was seen to result in a higher-order tetrameric arrangement (16, 20).

### Structural features of RNase J1 and RNase J2

The crystal structures of the catalytic domains of both RNase J1 and RNase J2 were determined. In both cases, the flexible C-terminal domain led to substantial sample heterogeneity. In the case of RNase J1, a C-terminal deletion construct (RNase J1 ΔCTD corresponding to 1–450 aa) could be crystallized. Although RNase J2 could be readily crystallized, analysis of the protein sample from a single crystal by MALDI-TOF MS suggested that a polypeptide (a 52-kDa fragment containing the catalytic domain) alone had crystallized and not the full-length RNase J2 that was used in the crystallization trials. It appears likely that the flexible C-terminal domain was proteolytically cleaved *in situ* by a protease contaminant that evaded the multiple protein purification steps. The diffraction data and refinement statistics for crystals of both paralogs are compiled in Table 1. Phase information to determine experimental electron density maps of RNase J1 and RNase J2 was obtained by molecular replacement using *B. subtilis* RNase J1 as a search model (PDB ID: 3ZQ4; 66 and 44% sequence identity with *S. epidermidis* RNase J1 and RNase J2, respectively) (20).

**Table 1:** Diffraction data, refinement, and model statistics

Parameters	RNase J1 (PDB: 6K6S)	RNase J2 (PDB: 6K6W)
Wavelength (Å)	0.966 Å	1.5418 Å
Resolution (Å)	50.58–2.99 (3.17–2.99)	36.39–2.56 (2.7–2.56)
Space group	P6_1_22	P12_1_1
Cell dimensions	a = b = 86.07 Å, c = 486.84 Å	a = 64.55 Å, b = 131.04 Å, c = 115.90 Å, β = 91.72°
R_sym_ (%)	9.5 (80.2)	14.3 (71.9)
Total number of reflections	133,065 (23,856)	265,384 (21,850)
Total number of unique reflections	21,554 (3405)	47,771 (3949)
Multiplicity	6.2 (7.0)	5.6 (5.5)
Mean I/σI	19.8 (1.4)	9.5 (3.0)
Completeness (%)	95.4 (96.2)	90.6 (81.8)
Anomalous completeness (%)	83.9 (85.0)	85.6 (76.3)
CC(1/2)	0.971 (0.764)	0.986 (0.744)
Solvent content (%)	49.97	47.82
Mosaicity (°)	0.76	1.12
Average B factors (Å^2^)	97	31.1
R_free_ (%)	32.90	25.46
R_work_ (%)	28.62	20.02
r.m.s. deviations		
Bonds (Å)	0.005	0.006
Angles (°)	1.06	0.93
Ramachandran outliers (%)	0.34	0.11
Ramachandran preferred (%)	92.95	97.00
Ramachandran allowed (%)	6.70	2.89
Number of nonH atoms	5848	13,403
Protein residues	884	1772
Metal ions	4	2
Water	11	113


Values for outer shells are given in parenthesis.

R_sym_ = Σ_j_|−I_j_|/Σ, where I_j_ is the intensity of the jth reflection and is the average intensity.

R_work_ = Σ_hkl_|F_o_ – F_c_|/Σ_hkl_|F_o_|.

R_free_ was calculated as for R_work_ but on 5% of the data excluded from the refinement calculation.

The overall structure of *S. epidermidis* RNase J1 and RNase J2 is similar to previously determined structures which include *B. subtilis* RNase J1 and other RNase J homologs from *T. thermophilus*, *S. coelicolor*, *D. radiodurans*, and *M. psychrophilus* (17, 20, 22, 23, 24). The catalytic domain could be modeled in both structures. This consists of the N-terminal β-lactamase (residues 4–200 and 370–432) and β-CASP domains (residues 206–360) (Fig. S3). The active site lies at the interface between the β-lactamase and β-CASP domain. A comparison of the active site cavity in these paralogs reveals that although the cavity shape is similar in all RNase J enzymes, the volume of the active site pocket differs; it is smaller in *S. epidermidis* RNase J2 (2096 Å^3^) when compared with *S. epidermidis* RNase J1 (3108 Å^3^) or *B. subtilis* RNase J1 (2649 Å^3^) (Table S1, Fig. S4, and Fig. S5). For this analysis, the cavity volume was calculated after cavity mapping using HOLLOW (25).

Because the structures of the two RNase J paralogs were determined in the apo form (without RNA), a putative RNA substrate was modeled to evaluate potential differences in substrate recognition. The RNA template used was the 5′ mono-phosphorylated 5-nucleotide from the *S. coelicolor* RNase J/RNA complex (PDB ID: 5A0T) (17). From this simplistic model of the RNase J1/RNA and RNase J2/RNA complexes, we note that residues that make interactions with nucleobases of RNA, starting from the site of entry of RNA to the active site of RNase J, are mostly conserved in the two *S. epidermidis* RNase J paralogs. We also note that the 5′-terminal phosphate interactions mediated by residues that connect the β-CASP to the β-lactamase domain (residues Pro-360 to Cys-372 in *S. epidermidis* RNase J2 and His-357 to Ser-370 in RNase J1) are similar to other characterized RNase J enzymes (Fig. S5, A and B) (17, 22, 23). For example, in the model of the RNase J1/RNA complex, the 5′-terminal nucleotide (C/0) is likely to be stabilized by contacts with His-368 and His-390 (Fig. S5, C and D). The structures and substrate models alone are thus inconclusive to understand why some RNase J paralogs (*B. subtilis* RNase J1, for example) specifically act on 5′ mono-phosphorylated substrates, whereas others are more liberal in substrate recognition (Fig. S5, D and E).

### The active site of S. epidermidis RNase J1 and RNase J2

**Figure 2:**
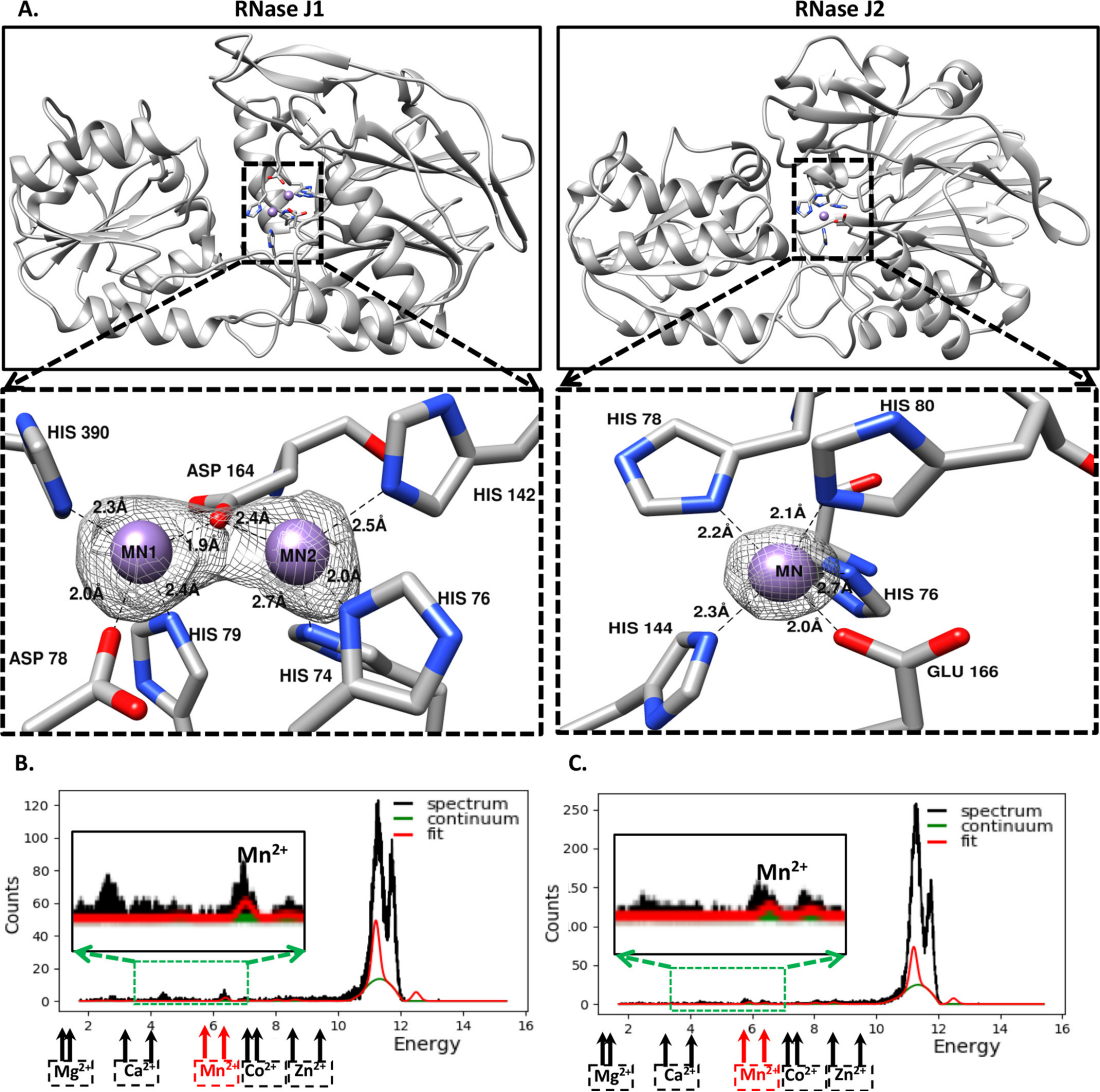
**Both RNase J paralogs require a metal cofactor for catalysis.***A*, RNase J1 has two metal ions at the active site. These bound metal ions could be modeled in the (2mFo-DFc) experimental electron density map at 5 σ level. Only one metal ion could be modeled at the active site of RNase J2. Modeling of these metal ions in the experimental electron density maps was guided by anomalous difference density calculations (Fig. S7). *B*, the X-ray fluorescence scan of an RNase J1 crystal and (*C*) an RNase J2 crystal confirms the presence of Mn^2+^ as the bound metal cofactor. The locations of different metal ions on the energy scale (keV) are marked on the fluorescence spectrum. The X-ray fluorescence scans thus suggest that these protein crystals contain the Mn^2+^-bound enzyme despite the likelihood of other metal cofactors (Fig. S10).

**Figure 3:**
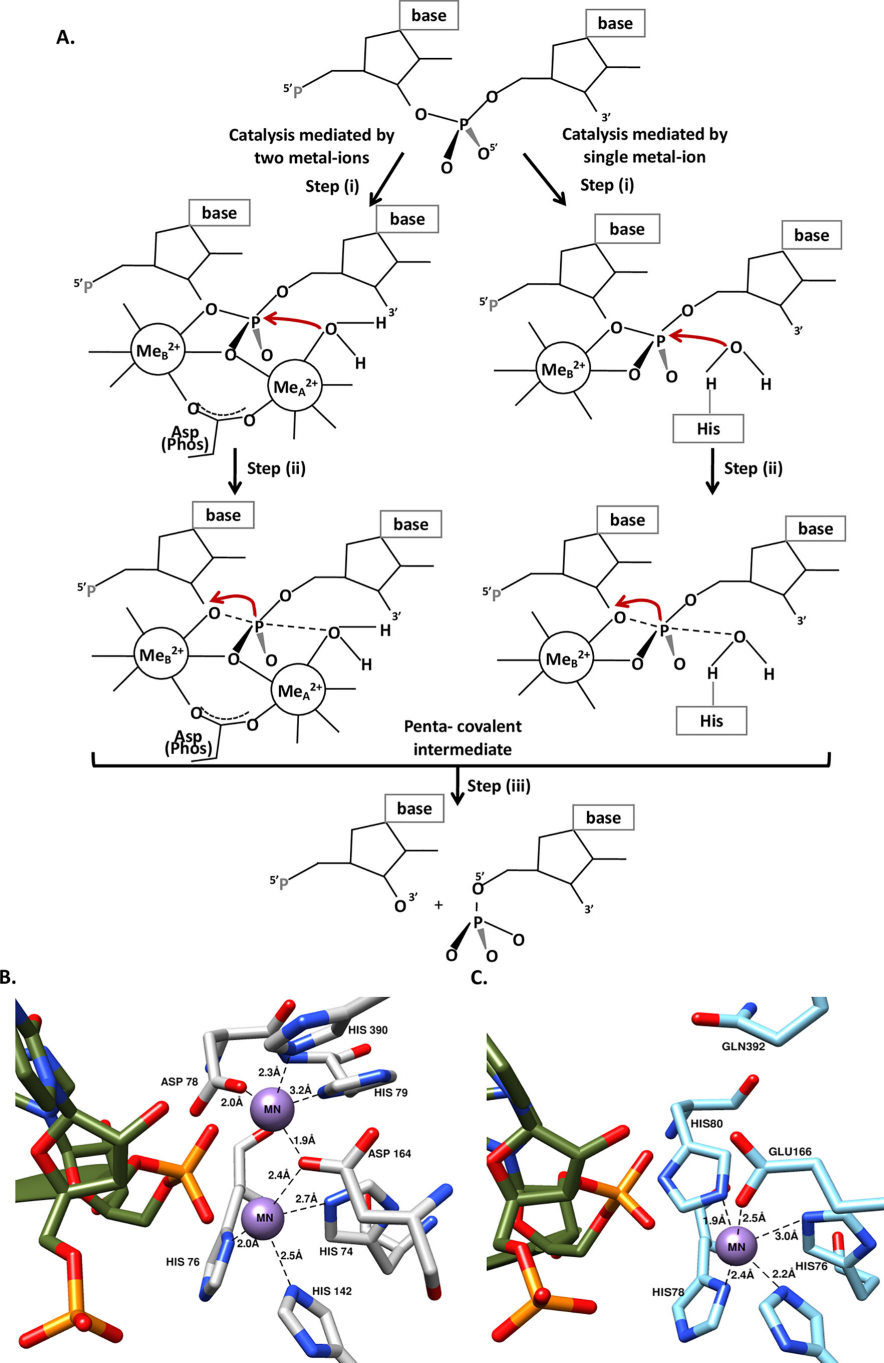
**Proposed reaction mechanisms of RNase J1 and RNase J2.** Schematic of the two-metal ion– (RNase J1) and single-metal-ion–dependent catalytic mechanism (RNase J2). *A*, essential steps in the metal-dependent catalytic mechanism include (i) activation of a bound water by two metal ions, (ii) attack of the generated nucleophile (hydroxyl ion) on the phosphorus of scissile phosphate leading to a negatively charged pentacovalent phosphate transition state, and (iii) cleavage of the phosphodiester bond followed by release of the product. *B*, coordination of the two metal ions in *S. epidermidis* RNase J1. *C*, coordination of the single metal ion in the active site of *S. epidermidis* RNase J2.

The crystal structure of the catalytic domain of *S. epidermidis* RNase J1 reveals two Mn^2+^ ions at the catalytic site (Fig. 2). The coordination geometry of the first bound Manganese ion is tetrahedral and is coordinated by residues Asp-78, His-79, Asp-164, and His-390. The second Mn^2+^ ion in RNase J1 has an octahedral coordination geometry involving His-74, His-76, His-142, and Asp-164. Although this finding (two metal ions) is similar to other characterized RNase J homologs, most other RNase J enzymes have two Zn^2+^ ions (Fig. S6, A and B). The crystal structure of RNase J2, on the other hand, revealed electron density for only one metal cofactor (Mn^2+^) at the active site (Fig. 2). In this case, the coordination geometry of the bound manganese ion is octahedral and is coordinated by residues His-76, His-78, His-80, His-144, and Glu-166. The modeling of the metal ion in the active site of RNase J2 was aided by the excellent anomalous signal in the data collected at the home source (representative difference anomalous difference Fourier electron density maps shown in Fig. S7). The similarity in the active site composition of RNase J1 with RNase Z (26) suggests that RNase J1 is a *bona fide* member of the ancestral RNase J family with a two metal ion–dependent catalytic mechanism (Fig. 3). As stated earlier, the metal ion–dependent catalytic activity of RNase J1 was confirmed by the finding that mutation of residues that coordinate the bound metal ions leads to an inactive enzyme. RNase J2 also relies on a metal ion for catalytic activity, a finding confirmed by mutational analysis (described in more detail in the later sections). It is worth noting, however, that differences between the two RNase J paralogs are not confined to the stoichiometry of the metal ion at the active site. Structural features and sequence comparison of *S. epidermidis* RNase J2 reveal several differences in some of the key residues lining the active site (Fig. S6C). For example, Asp-78, His-79, Asp-164, and His-389 in RNase J1 are replaced by His-80, Ala-81, Glu-166, and Gln-392 at structurally equivalent positions in RNase J2 (Fig. S6C). These substitutions in the active site of *S. epidermidis* RNase J2 make it distinct from other characterized RNase J enzymes. As stated earlier, all RNase J structures solved thus far (homologs from *S. coelicolor*, *D. radiodurans*, *M. psychrophilus*, and *B. subtilis*) have two metal ions at the active site. This observation formed the basis for a two metal ion–dependent catalytic mechanism (17, 22, 23). Catalytic activity measurements for most of these enzymes were performed in the presence of either Mg^2+^or Mn^2+^ (16, 18, 20). Whereas *S. coelicolor* RNase J required Mg^2+^ for catalytic activity (17), *D. radiodurans* and *M. psychrophilus* RNase J preferred Mn^2+^ as the metal cofactor (27). *S. aureus* RNase J1 was also shown to require Mn^2+^ as the metal cofactor for catalytic activity (19).

Metal cofactors are crucial for the generation of a hydroxyl ion in RNase J enzymes (27). In the two-metal-ion cofactor mechanism, one metal ion helps in the generation of an activated water molecule (Fig. 3). The second metal ion stabilizes the negatively charged pentacovalent phosphate transition state. In the single-metal-ion–dependent mechanism, however, a histidine acts as a base and deprotonates a water molecule to generate the nucleophile (a hydroxyl ion). In this case, the metal ion plays a structural role in stabilizing the transition state of pentacovalent phosphate. These two characterized mechanisms provide a template to rationalize the catalytic mechanism in the *S. epidermidis* RNase J paralogs (Fig. 3). Together, comparison of the active site architecture thus suggests that RNase J1 is closer to “canonical” RNase J than RNase J2.

### RNase and DNase activity of RNase J1 and RNase J2

**Figure 4:**
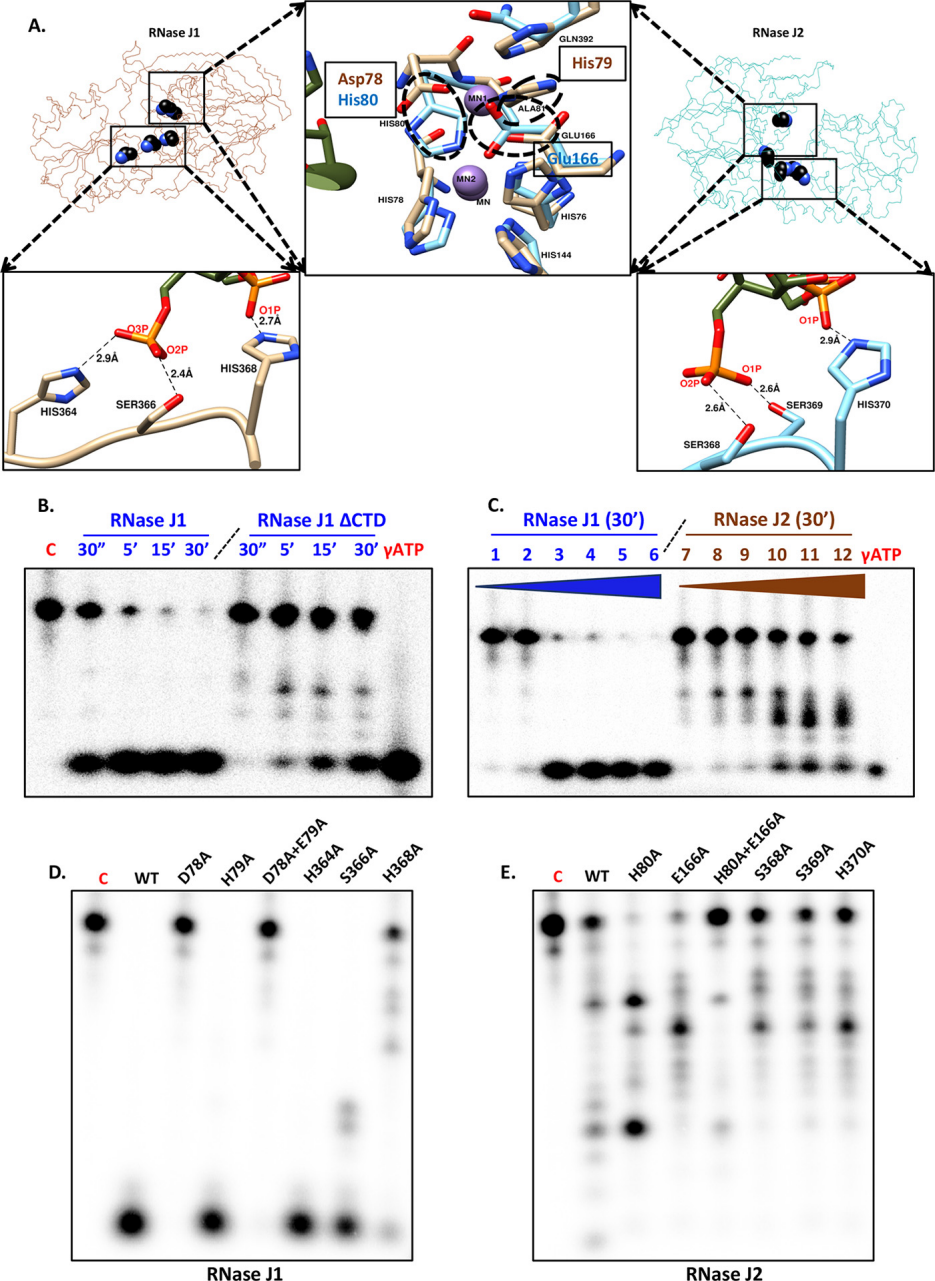
**Enzyme assays and mutational analysis of RNase J1 and RNase J2.***A*, the crystal structures of RNase J1 and RNase J2 suggested residues that were likely to be important for catalytic activity and substrate interactions. The locations of the sites that were selected for mutational analysis in the structure of RNase J1 and RNase J2 are highlighted. *B*, RNase J1 is primarily an exonuclease. The mobility of a 20-mer single-stranded RNA (5′-ACUGGACAAAUACUCCGAGG-3′) substrate on a 20% urea-PAGE was monitored in this experiment. The four *lanes* refer to representative time points after the reaction was initiated. The truncated RNase J1 (RNase J1 ΔCTD) enzyme is an endoRNase with minimal exoRNase activity. *C*, RNase J2 has substantially lower activity than RNase J1. This paralog has both endoRNase and exoRNase activity. This is evident from an experiment performed with increasing enzyme concentration at a fixed time point (30 min from the start of a reaction). The different lanes in the urea-PAGE are 1) 100 nm, 2) 300 nm, 3) 600 nm, 4) 1.2 µm, 5) 3.0 µm, and 6) 6 µm of RNase J1; and 7) 1.2 µm, 8) 3.0 µm, 9) 6 µm, 10) 48 µm, 11) 72 µm, and 12) 100 µm of RNase J2. These samples were evaluated at a fixed time point (30 min) after the reaction was initiated. *D*, activity of RNase J1 (WT enzyme) compared with that of the active site mutants (residues Asp-78 and His-79, labeled on the lanes of the gel for clarity) and site-specific mutants of residues that are potentially involved in phosphate recognition (H364A, S366A, and H368A). These enzyme assays were performed using a 27-mer RNA substrate (5′-UCUUUACGGUGCUAUUUUGUUUUGUUC-3′) used in the characterization of *S. aureus* RNase J1 described earlier. Although mutations of residues involved in metal ion coordination lead to an inactive enzyme, mutations of residues predicted to be involved in substrate recognition (especially Ser-366 and His-368) show impaired catalytic activity. *E*, activity of RNase J2 (WT enzyme) compared with that of the active site mutant (H80A and E166A) suggests that mutations of residues involved in metal ion coordination influence catalytic activity. Mutations of residues involved in phosphate recognition (Ser-368, Ser-369, and His-370) do not significantly alter catalytic activity. The 27-mer RNA substrate used in this experiment is the same as that employed for RNase J1 (*panel D*). Please refer to “Experimental procedures” for experimental details and composition of the reaction mixture.

The structural information on RNase J1 and RNase J2 aided the design of experiments to evaluate the catalytic activity of these paralogs and mutant enzymes (Fig. 4*A*). Assays to monitor endo- and exoRNase activity were performed using protocols described earlier with few modifications (19). In these assays, the upper band in the 20% urea-PAGE corresponds to an intact 20-mer RNA, whereas the band at the bottom corresponds to the product after 5′-exoRNase activity (Fig. 4*B*). In the case of RNase J1 (0.6 µm concentration), no product corresponding to endoRNase activity was seen in this reaction condition. When the activity assays for RNase J2 were performed at the same concentration as that for RNase J1, no product corresponding to either exo- or endoRNase activity was seen. This observation is similar to that reported for *B. subtilis* and *E. faecalis* RNase J2, which suggests that this paralog has substantially lower enzymatic activity compared with RNase J1 (14, 18). These assays were subsequently performed at a higher enzyme concentration (72 µm). At this concentration of RNase J2, we note both endoRNase activity and exonuclease activity (albeit significantly lower than RNase J1 exoRNase activity; Fig. 4*C*).

To evaluate whether the C-terminal deletion construct employed in the structural analysis of RNase J1 is functionally active, enzyme assays were performed with the same protein preparation used in the crystallization trials (RNase J1ΔCTD; corresponding to residues 1–450). These assays were performed with the same concentration of the enzyme and substrate (600 nm RNase J1 and 0.25 nm of the 20-mer RNA substrate) as that of the full-length RNase J1. We note that RNase J1ΔCTD is monomeric (Fig. 1*B*) and also has endoRNase activity (presence of bands between 20-mer RNA and mono-ribonucleotides) in addition to exoRNase activity (Fig. 4*B*). Together, the activity assays suggest that whereas full-length RNase J1 functions primarily as an exoRNase, RNase J1ΔCTD has both endoRNase and exoRNase activity.

To rule out the possibility for RNase activity (especially endoRNase activity) arising from a contaminant in enzyme preparations, activity assays were performed with active site mutants of RNase J1 and RNase J2. In the case of RNase J1, the D78A mutation led to an inactive enzyme (Fig. 4*D*). In the case of RNase J2, the H80A,E166A double mutant showed substantially impaired catalytic activity (Fig. 4*E*). To enable a comparison with enzyme assays performed on *S. aureus* RNase J1 (98% identity with *S. epidermidis* RNase J1), enzyme activity was evaluated using a 27-mer RNA substrate reported earlier (19). DNase activity of RNase J1 and RNase J2 was also monitored using a 27-mer DNA substrate (19). The results from these assays are presented in Fig. S8. We also note that DNase activity is more pronounced in the presence of Mn^2+^ when compared with Ca^2+^ for both RNase J1 and RNase J2 (Fig. S8, A and B). These enzymes were also seen to be active as an exonuclease (RNase J1) and endonuclease (RNase J2) with the DNA substrate. The cleavage pattern of J2 on the DNA substrate, however, is different. Furthermore, we note that Ca^2+^ switches RNase J1 from dominant exonuclease activity to an endonuclease on the RNA substrate alone. In the case of RNase J2, Ca^2+^ changes the cleavage pattern on the RNA substrate and abolishes DNase activity. A caveat on the interpretation of the apparent changes in cleavage specificity is that this could simply be a reflection of multiple rounds of enzyme activity on progressively shorter RNA fragments with the result that a more active enzyme would lead to shorter final end-products. Together, these enzyme assays reveal that the *S. epidermidis* RNase J paralogs can perform RNase and DNase activity.

### Validation of functionally relevant residues identified from the crystal structures

The crystal structure of RNase J1 suggested that residues His-364, Ser-366, and His-368 could be potentially involved in phosphate recognition (Fig. S5). Whereas mutation of His-364 did not appear to influence catalytic activity, mutations of the two other residues predicted to be involved in substrate recognition (Ser-366 and His-368) showed impaired catalytic activity (Fig. 4*D*). The finding that the RNase J1 H368A mutant has substantially lower catalytic activity than the WT enzyme is particularly significant because this is consistent with the hypothesis that His-368, located in the loop connecting the β-lactamase and β-CASP domains, plays an important role in catalysis (Fig. 4*D* and Fig. S5D). The loss in catalytic activity in the H368A mutant is likely due to impaired phosphate binding. Previous reports on the characterization of this enzyme suggest that RNase J1 could efficiently degrade a triphosphorylated RNA (24). The preference for 5′ monophosphate or 5′ triphosphate in the RNA substrate varies from about 3- to almost 10-fold across RNase J enzymes characterized thus far. The substantial loss in the catalytic activity of the RNase J1 H368A mutant enzyme suggests that difference in the substrate preference (5′ monophosphate or 5′ triphosphate) can potentially be correlated with the positioning of this histidine residue in different RNase J paralogs. This suggestion is consistent with the finding that mutating residues in structurally similar positions in RNase J2 (Ser-368, Ser-369, and His-370) also lowered enzyme activity (Fig. 4*E*).

### Metal cofactor preferences differ for RNase J1 and RNase J2

The catalytic activity of *S. epidermidis* RNase J1 was evaluated in the presence of different metal ions (Mn^2+^, Mg^2+^, Ca^2+^, Co^2+^, and Zn^2+^) (Fig. S9). Presence of the metal ion in freshly purified enzyme samples was evaluated by inductively coupled plasma MS (ICP-MS) analysis (Fig. S10). We note that Mn^2+^ is the preferred metal cofactor for this enzyme, a finding that is consistent with the observations made by Hausmann *et al.* (19), in the case of *S. aureus* RNase J1. Mg^2+^ and Co^2+^ also facilitate catalytic activity, albeit to a lesser extent than Mn^2+^. Ca^2+^ and Zn^2+^ failed to elicit RNase J1 activity altogether. In a competition experiment, Mn^2+^ was kept constant (1 mm) and competing metal ions (2 mm) were added to the reaction mixture (Fig. S9, A and B). These experiments suggest that Mn^2+^ and Mg^2+^are preferred metal cofactors for catalytic activity.

In the case of enzyme assays for RNase J2 performed in the presence of different metal ions, maximal activity was seen in the presence of Ca^2+^, followed by Mg^2+^ and Mn^2+^, whereas Co^2+^ and Zn^2+^ cofactors elicited negligible catalytic activity (Fig. S9C). This finding is consistent with the results from the competition assays where Mn^2+^ was kept constant (1 mm) and different metal ions (Mg^2+^, Ca^2+^, Co^2+^, and Zn^2+^) were added (2 mm) to the reaction mixture (Fig. S9D). To further confirm the observation that Ca^2+^ was a preferred metal cofactor for RNase J2, activity assays were performed wherein Ca^2+^ was kept constant (1 mm) and other metal ions (Mg^2+^, Mn^2+^, Co^2+^, and Zn^2+^) were added at a higher concentration (2 mm) (Fig. S9E). The replacement of the metal cofactors in the enzymes used in these activity assays was also evaluated by ICP-MS analysis (Fig. S10). It is worth noting in this context that whereas ICP-MS data confirm metal ion substitution, the stoichiometry and binding site(s) involved in Ca^2+^ binding remains to be determined.

### Expression levels of RNase J paralogs vary across staphylococcal strains

**Figure 5:**
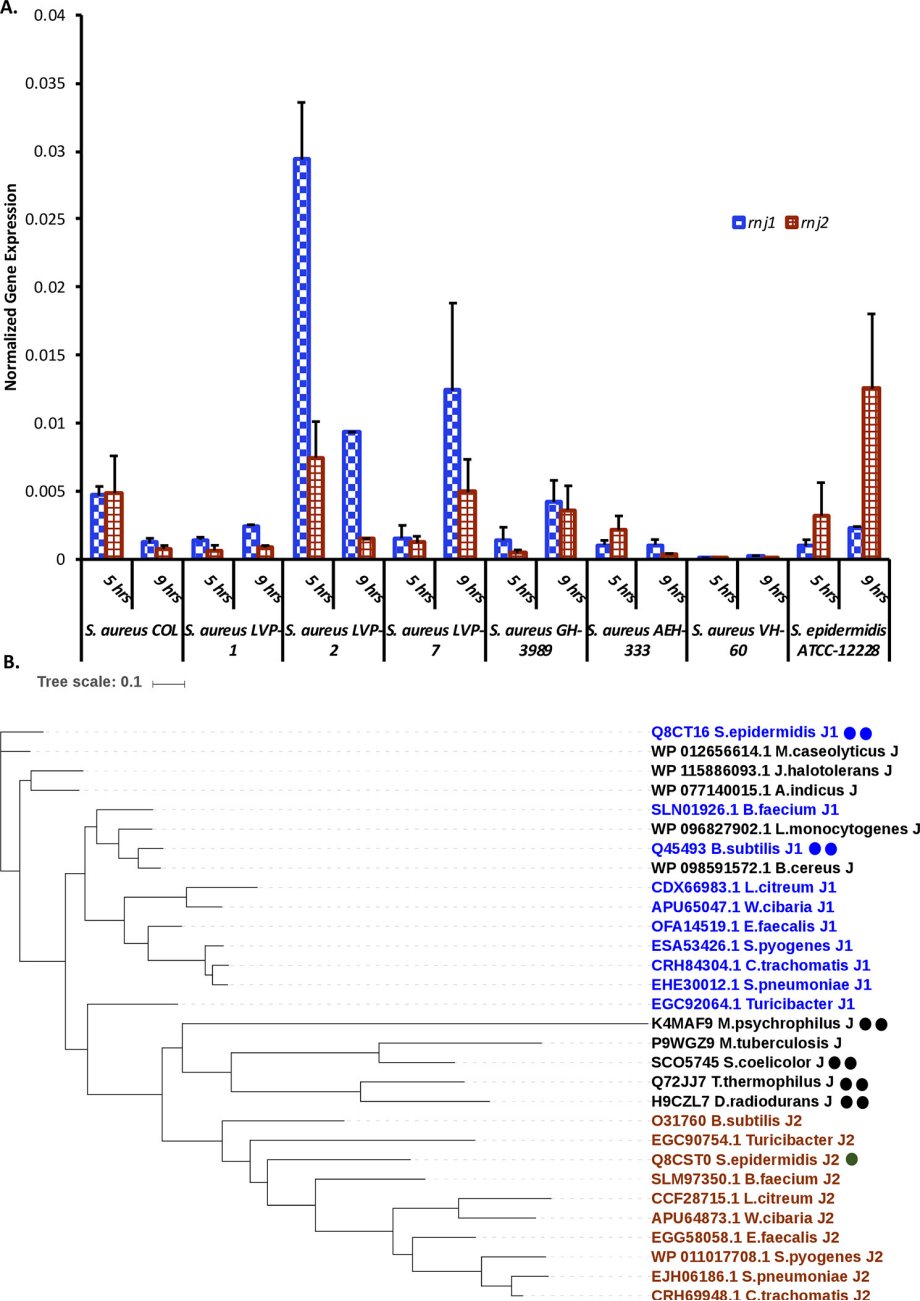
**Multiple RNase J paralogs provide a potential route to functional diversification.***A*, the expression levels of the two RNase J paralogs vary across different *Staphylococcal* strains. The data shown represent an average of six measurements (two biological replicates). Details of the strains including the presence of representative phage signatures in the genomes of these strains are presented in Fig. S11. *B*, phylogenetic analysis reveals that RNase J1 is closer to RNase J than RNase J2. The number of metal ions at the active site is represented alongside the annotation of enzymes in the dendrogram.

In the case of *B. subtilis*, the genes encoding the two RNase J paralogs (*rnjA* and *rnjB*) lie in different operons. However, the expression of *rnjA* (encoding RNase J1) was not found to be at levels significantly different from *rnjB* (encoding RNase J2). This finding suggested that RNase J1 does not exist alone to a significant extent. This inference is consistent with the observation that the *B. subtilis* paralogs RNase J1 and RNase J2 interact with 1:1 stoichiometry (16). Because the genes encoding these two enzymes (*rnj1* and *rnj2*) also belong to different operons in *Staphylococci*, it was important to quantify the expression levels of these genes. One caveat of this inference is that expression level of the genes translates to corresponding changes in the intracellular level of these proteins. To evaluate expression levels of *rnj1* and *rnj2*, we quantified the transcript levels of both *rnj1* and *rnj2* at two time points (log and stationary phase) in eight representative *Staphylococcal* strains (Fig. 5*A*). These strains were chosen to represent different lineages, nosocomial and community backgrounds (Fig. S11). Alongside the diversity in the lineages of these *Staphylococcal* strains, we also examined the prophage signatures in these *Staphylococcal* strains using an online tool (Fig. S11) (28). One observation from the qRT-PCR data is that the expression of these two RNase J paralogs can vary in different *Staphylococccal* strains (Fig. 5*A*). For example, the expression levels of *rnj1* at the exponential phase of growth in the LVP2 strain are higher when compared with other clinical *S. aureus* strains, including COL (Fig. 5*A*). The LVP2 strain belongs to the ST772 lineage (29). We also note that the expression levels of *S. epidermidis rnj2* are higher when compared with *rnj1*.

## Discussion

Two prominent differences between the structures of the two RNase J paralogs are the active site architecture and the stoichiometry of the metal cofactor at the active site. The structures reveal that the RNase J1 active site is closer to other characterized RNase J enzymes or even eukaryotic MRE-11 with two Mn^2+^ ions at the active site (30). Indeed, a major difference from previous structural descriptions of RNase J enzymes is that both *S. epidermidis* enzymes, RNase J1 and RNase J2, have Mn^2+^ at the active site. The arrangement of two metal ions in RNase J1 is functionally significant (Fig. 3). In the substrate-bound form of RNase H, for example, of the two metal ions at the active site, an irregular pentacovalent coordination for one metal cofactor was suggested to drive catalysis. The suggestion in this case was that because this catalytically relevant metal cofactor (Mg^2+^_B_) seeks to reorient from an irregular pentacoordinated arrangement to the preferred octahedral geometry, the enzyme-substrate complex is destabilized to drive catalysis (31). In this context, Ca^2+^ is considered incompatible with two-metal cofactor catalysis because this cation can accommodate multiple coordination geometries. Indeed, Ca^2+^ is used to generate inactive enzyme–substrate complexes (31). Structural studies on other RNase J homologs reveal that whereas one metal cofactor participates in catalysis, the other aligns the nucleophile with the scissile phosphate. It is worth noting in this context that whereas the crystal structure of RNase J2 revealed a Mn^2+^-bound enzyme (also confirmed by the X-ray fluorescence scan, Fig. 2*C*), enzyme assays suggest that RNase J2 prefers Ca^2+^ for its catalytic activity *in vitro.* A related observation is that mutating residues that coordinate the bound metal ion in RNase J2 (His-80 and Glu-166) influence enzyme activity (Fig. 4*E*). It thus appears likely that the metal cofactor plays a more structural role in this enzyme as opposed to direct involvement in the catalytic mechanism. It however remains to be determined whether RNase J2 is bound to Mn^2+^ or Ca^2+^ *in vivo*. In this context, a phylogenetic analysis of RNase J paralogs also provided an interesting insight (Fig. 5*B*). RNase J2 paralogs appear to be phylogenetically distinct; whether other RNase J2 paralogs have a similar active site architecture characterized by one metal cofactor remains to be determined. Together, the structural and biochemical data on *S. epidermidis* RNase J1 and RNase J2 reveal that (i) oligomerization is an important determinant in the switch between exo- and endoRNase activity; (ii) the metal cofactor and stoichiometry determine the reaction mechanism; this difference is hardwired in RNase J paralogs; (iii) RNase J1 is phylogenetically closer to ancestral RNase J than RNase J2; and (iv) the expression levels of these paralogs vary across different *Staphylococcal* strains.

The structural data on the *S. epidermidis* RNase J paralogs also provide a rationale to understand observations made on the *B. subtilis* RNase J1 and RNase J2 enzymes. Similarity in RNA substrate recognition, for example, can be rationalized from the observation that residues in the monophosphate interaction site are conserved in both RNase J1 and RNase J2 (Fig. 4 and Fig. S5). Another important observation is that the signature HxHxDH motif in the β-lactamase domain is not retained in RNase J2 (32). Indeed, experiments on *B. subtilis* RNase J2 demonstrate that mutating this signature motif to match the one present in RNase J1 abolished residual 5′exoribonuclease activity (16).

The bacterial RNase J–specific C-terminal domain appears to be an important part of the evolutionary diversification. In the case of *B. subtilis* RNase J1, for example, the extensive interaction surface involving the C-terminal domain drives dimerization. The finding that RNase J2 is monomeric thus suggests that minimal changes in the C-terminal domain can alter the quaternary structure in RNase J enzymes. Apart from the fact that the active site architecture of RNase J2 is distinct from RNase J1, the finding that exoRNase activity is more associated with quaternary structure (dimer in the case of RNase J1) is relevant. The C-terminal segment that governs oligomeric assembly is thus an important determinant of the switch between exo- and endoRNase activity.

The functional and evolutionary rationale for the *Staphylococcal* RNase J paralogs is also intriguing in the light of some recent findings: RNase J2 has been suggested to potentially serve as a stabilizer of RNase J1 in *S. aureus* (13). The type III CRISPR-Cas multisubunit effector complex in *S. epidermidis* utilizes RNase J2 and PNPase for degradation of phage DNA and RNA (33). Indeed, it was seen that RNase J2 copurifies with the Cas10-Csm complex (34). In this study, it was shown that RNase J2 prevents the transfer of conjugate plasmids leading to an effective defense mechanism against phages. Furthermore, PNPase and RNase J2 significantly restricted phage DNA amplification within the cell even in the absence of the CRISPR system. Although only a limited number of clinical strains were examined in this study, these represent different lineages (PHAST database (28); Fig. S11). Albeit preliminary, the finding that the expression levels of *rnj1* and *rnj2* vary across *Staphylococcal* strains could potentially be relevant. For example, the expression levels of *rnj1* are much higher in a clinical *S. aureus* strain (LVP2) when compared with the other strains that were examined (Fig. 5*A*).

Put together, the *S. epidermidis* RNase J paralogs suggest functional optimization utilizing differences in active site architecture and preferred quaternary arrangement. Whereas RNase J1 is closer to the canonical RNase J (dimeric arrangement, two metal ion–based catalytic activity, 5′ base–dependent exoRNase activity), RNase J2 is distinct as a monomeric enzyme with a single Mn^2+^ ion at the active site. Because the genes encoding these two enzymes are not part of the same operon, changes in the expression level of these paralogs could, in effect, form a regulatory mechanism. RNase J paralogs in *Staphylococci* thus represent an example of a multi-layered regulatory mechanism enabling functional diversity, ranging from phage immunity, RNA maturation, RNA recycling, and changes in gene expression.

## Experimental procedures

### Cloning and purification

The genes encoding RNase J1 (amino acids 1–560), the C-terminal domain deletion construct of RNase J1 (residues 1–450, hereafter referred to as RNase J1ΔCTD), and RNase J2 were cloned in Multiple Cloning Site (MCS) I of the pETDuet-1 expression vector between the BamHI and HindIII restriction sites (Table S2). The recombinant enzymes have a 6× His tag at the N terminus. Mutations of specific residues involved in metal ion coordination or the catalytic mechanism in RNase J1 were made using the double primer method. The list of site-specific mutations in RNase J1 and RNase J2 is compiled in Table S3. To overexpress the recombinant enzyme, the desired plasmids were transformed in *E. coli* BL21 Star (DE3) strain (Novagen). A single transformant colony was inoculated in Luria Bertani broth containing 50 µg/ml ampicillin. *E. coli* cultures were induced with 0.5 mm isopropyl β-d-1-thiogalactopyranoside at an *A*_600nm_ of 0.5. The cells were further grown for 12 h at 18 °C before harvesting by centrifugation.

Proteins were purified using immobilized metal affinity chromatography. Briefly, pellets were lysed in buffer A (25 mm Tris-HCl, pH 8.0, 250 mm NaCl, 0.5 mm MnCl_2_, 2 mm β-mercaptoethanol (β-ME), and 10% glycerol) in the presence of 2 mm PMSF. After centrifugation, the supernatant was incubated with nickel-nitrilotriacetic acid resin. A high salt wash (1.5 m NaCl in buffer A) with ∼10 column volumes helped in the removal of bound nucleic acid contaminants. The recombinant protein bound to the resin was then eluted stepwise with imidazole concentrations ranging from 250–500 mm in buffer A. The eluted proteins were further subjected to size exclusion chromatography using a Hi-prep 16/60 Sephacryl S-200 column (GE Healthcare) in buffer B (25 mm Tris-HCl, pH 8.0, 250 mm NaCl, 0.5 mm MnCl_2_, 2 mm β-mercaptoethanol, 0.5 mm tris(2-carboxyethyl)phosphine, and 10% glycerol).

### Coexpression and copurification of RNase J1 and RNase J2

The plasmid containing *rnj*2 cloned in the MCS-I of the pETDuet-1 expression vector was used as a backbone to insert *rnj1* in MCS II between the *MfeI* and XhoI restriction sites (Table S2). In this construct, recombinant RNase J2 had a 6×-His tag at the N terminus. The recombinant enzymes were over expressed in *E. coli* BL21 Star (DE3) strain (Novogen, Inc). A single transformed colony was inoculated in Luria Bertani broth containing 50 µg/ml ampicillin. *E. coli* cultures were induced with 0.5 mm isopropyl β-d-1-thiogalactopyranoside at an *A*_600nm_ of 0.5. The cells were further grown for 12 h at 18 °C before harvesting by centrifugation.

The coexpressed proteins were copurified by immobilized metal affinity chromatography. Briefly, the cells were lysed in lysis buffer containing 50 mm Tris, pH 8.0, 250 mm NaCl, 2 mm β-ME, 10% glycerol, and 2 mm PMSF. After centrifugation, the supernatant was incubated with nickel-nitrilotriacetic acid resin. The recombinant protein bound to the resin was then eluted stepwise with imidazole concentrations ranging from 100–400 mm. The eluted protein was digested with 20 µg/ml trypsin (Sigma-Aldrich) and the fragments were analyzed by MS (MALDI-TOF; Bruker Daltonics).

### Quaternary structure of the RNase J paralogs and mutant enzymes

Analytical size exclusion chromatography was used to analyze the oligomeric state of the purified recombinant enzymes. In these experiments, 250 μg of freshly purified RNase J1, RNase J1ΔCTD, and RNase J2 were injected separately into a Superdex200 column (GE Healthcare) equilibrated in 50 mm Tris-HCl, pH 8.0, containing 250 mm NaCl, 10% glycerol, 4 mm β-ME, 0.5 mm tris(2-carboxyethyl)phosphine, and 5 mm MgCl_2_. The same size exclusion column and identical buffer conditions were maintained for SEC-MALS experiments performed on the two RNase J paralogs.

### Crystallization and structure determination of RNase J1 and RNase J2

Crystallization trials were performed at different concentrations (4 mg/ml, 6 mg/ml, and 8 mg/ml) of the truncated RNase J1 enzyme (comprising residues 1–450). Sparse-matrix sampling using commercial crystallization screens (Hampton Research and Jena Biosciences) was performed with freshly purified and concentrated protein samples (in 25 mm Tris-HCl, pH 8.0, 250 mm NaCl, 2 mm β-mercaptoethanol, and 10% glycerol). Crystallization trials were performed using both microbatch and hanging-drop methods. Although crystalline precipitates were obtained in a variety of conditions, the best diffracting crystals of RNase J1 were obtained using the microbatch method in a condition containing 10 mm citric acid, 70 mm bis-tris propane, pH 9.3, 20% w/v PEG 3350, and 0.1 m spermine tetrahydrochloride. A typical 2-µl drop contained protein:crystallization condition in a 1:1 ratio. The protein crystals were cryoprotected as-is without soaking in any metal ion–containing buffer. All crystallization experiments were performed at 20 °C. Diffraction data were collected at the ID30A-1 beamline of the European Synchrotron Radiation Facility, Grenoble, France. The diffraction data were integrated using *iMosflm* and scaled using *SCALA* (35, 36). Phase information was obtained by molecular replacement, performed using PHASER (37) with *B. subtilis* RNase J (PDB: 3ZQ4) as a search model. The model corresponding to RNase J1 was further built using *Coot* and refined using REFMAC and Phenix (36, 38, 39). Structural illustrations were prepared using Chimera (40).

In the case of RNase J2, crystallization trials were performed using the microbatch method at a protein concentration of 10 mg/ml in 25 mm Tris-HCl, pH 8.0, 250 mm NaCl, 2 mm β-mercaptoethanol, and 10% glycerol. The initial crystallization condition for RNase J2 contained 100 mm trisodium citrate dihydrate, pH 5.6, 20% isopropanol, and 20% PEG 4000. These crystals diffracted to low resolution (∼10 Å). To improve crystal quality, additives (Hampton Research) were screened in the ratio of 2:2:0.4 (condition:protein:additive) alongside seeding (both micro-seeding and streak seeding). Diffracting crystals were obtained after addition of 40% formamide and 1 m lithium chloride. RNase J2 crystals diffracted beyond 2.6 Å with 10 min of exposure at the home source (Rigaku FRE X-ray generator and R-axis VI detector). As in the case of RNase J1, the diffraction data were integrated using *iMosflm* and scaled using *SCALA* (35, 36). Phase information was obtained by molecular replacement performed using PHASER (37) with *B. subtilis* RNase J (PDB: 3ZQ4) as a search model. The model was rebuilt using *Coot* and refined using REFMAC and Phenix (36, 38, 39). All structural illustrations were prepared using Chimera (40).

### Phylogenetic analysis of RNase J1 and RNase J2

Multiple sequence alignment of 30 RNase J sequences with sequence identities ranging from 28% to 85% with query coverage of greater than 75% was performed. This sequence data set contained 10 annotated RNase J1, RNase J2 and RNase J sequences. The multiple sequence alignment was performed using Clustal Omega (41). The phylogenetic tree was constructed using IQ-TREE wherein Blosum62 and Gamma (+G) 4 were used with 10,000 ultrafast bootstrap alignments (41). The maximum correlation coefficient used in this analysis was 0.9. (iTOL)v3 was used to display and annotate the phylogenetic tree (42).

### Analysis of the active site cavity in structurally characterized RNase J

The active sites of RNase J1 and RNase J2 were visualized using a protocol implemented in HOLLOW (25). To ensure uniformity across different structures, the seed point (reference point for the cavity calculation) was kept constant. For example, in *S. epidermidis* RNase J1, ND1 of His-76 (His-78 in RNase J2) was selected as the seed point, and the cavity profile was generated with 12 Å of radius around this seed point. Similarly, the cavity shape profile was generated taking the equivalent atom position with respect to the ND1 of His-76 in other RNase J homologs.

### Molecular model of RNA interactions

To understand RNA-protein interactions in the two RNase J paralogs, RNA coordinates were ported from the crystal structure of *S. coelicolor* RNase J (PDB ID: 5A0T). Structures of RNase J1 and RNase J2 were superposed on the *S. coelicolor* homolog to obtain an initial model with a putative RNA substrate (40). The model of the complex was energy minimized using assisted model building with energy refinement force field in Gromacs (43). NUCPLOT (44) was used to visualize the enzyme-RNA interactions.

### Catalytic activity of RNase J1 and RNase J2

Enzyme assays for RNase J1, RNase J2, and the C-terminal deletion constructs were performed in a buffer containing 50 mm Tris-HCl, pH 8.0, 100 mm KCl, 5% glycerol, and 0.6 mm MnCl_2_. RNase activity was evaluated using two substrates, *viz*. a 20-mer single-stranded RNA (5′-ACUGGACAAAUACUCCGAGG-3′) and a 27-mer single-stranded RNA (5′-UCU-UUACGGUGCUAUUUUGUUUUGUUC-3′) (19). DNase activity of RNase J1 and RNase J2 was evaluated using a 27-mer DNA substrate (5′-TCTTTACGGTGCTATTTTGTTTTGTTC-3′). For these assays, the single-stranded oligonucleotide substrates were radiolabeled at the 5′-end using radioactive ^32^P (γATP). After radiolabeling using polynucleotide kinase (New England Biolabs), the radiolabeled oligos were purified by ethanol precipitation (45). The activity assays were performed at 37 °C, and the products were evaluated at different time point intervals. Equal amounts of reaction samples were examined at 0.5, 5, 15, and 30 min from the start of the reaction. The reaction was stopped by the addition of RNA loading dye and samples were loaded on a 20% urea-PAGE. These gels were dried and subsequently scanned using a phosphorimager (Bio-Rad). The activity levels of RNase J1 and the C-terminal deletion construct (RNase J1ΔCTD) was compared under identical conditions (reaction mixture contained 600 nm of enzyme and was evaluated at specific time points 0.5, 5, 15, and 30 min). The RNase activity levels of RNase J1 and RNase J2 was compared at different concentrations of RNase J1 (100 nm, 300 nm, 600 nm, 1.2 µm, 3.0 µm, and 6 µm) and RNase J2 (1.2 µm, 3.0 µm, 6 µm, 48 µm, 72 µm, and 100 µm). For ease in comparison, we evaluated RNase activity at a fixed time point (30 min). In an effort to determine the most preferred metal ion for RNase J1 and RNase J2 catalytic activity, assays were performed in the presence of different divalent cations (1 mm each of Mn^2+^, Mg^2+^, Ca^2+^, Co^2+^, and Zn^2+^). Apo enzymes were prepared by incubating freshly purified enzyme with a chelator (0.001 m EDTA) followed by extensive dialysis. ICP-MS analysis was used to evaluate the presence of the bound metal ion. In these metal ion competition experiments, the concentration of one metal ion was maintained at a constant level (1 mm) and the potential competing metal ion (at a higher concentration, 2 mm) was added to the reaction mixture.

### Evaluation of staphylococcal strains for expression of rnj1 and rnj2

The details of the *Staphylococcal* strains used in this study are listed in Fig. S11. *S. epidermidis* (ATCC 12228), *S. aureus* COL (kind gift from Hermenia de Lancastre, Rockefeller University, NY), and characterized *S. aureus* strains belonging to a different sequence type from those in our collection (29, 46) were examined. 1% inoculum from an overnight grown culture was inoculated into fresh Tryptone Soya Broth (HiMedia), and the absorbance at 600 nm was recorded every hour in a microplate reader (SpectraMax M3). 0.6 ml of harvested cells from different growth phases (exponential and stationary) were mixed with two volumes of RNAprotect® Bacteria Reagent (Qiagen), incubated for 10 min at room temperature, and centrifuged at 7,000 rpm for 10 min at 4 °C. These cells were flash-frozen and stored at −80 °C until further use. The relative levels of expression of the RNase J paralogs were determined by using the 2^−ΔCT^ method (47).

### RNA isolation, cDNA synthesis, and qRT-PCR analysis

Total RNA samples were extracted from different growth phases (exponential and stationary) using RNeasy mini kit (Qiagen). Initially, the cells were lysed in 30 mm Tris, pH 8.0, 1 mm EDTA containing 100 µg of lysozyme (Sigma-Aldrich), 200 µg of Proteinase K (Qiagen), and 100 µg of lysostaphin (Sigma-Aldrich). No changes were made to the protocol suggested by the manufacturer. cDNA was prepared using QuantiTect® Reverse Transcription kit (Qiagen) according to the manufacturer's instructions. Real-time PCR was carried out for samples from different strains (exponential and stationary phase RNA samples were evaluated) to quantitate the levels of *rnj1* and *rnj2* using SYBR green super mix on a Bio-Rad iQ5^TM^ thermo cycler. 16S rRNA was used as internal control in these experiments (48). The primers used to amplify the target genes are listed in Table S4.

## Data availability

The data produced by this study are available in this article and in the supporting information.

The atomic coordinates and structure factors (6K6S and 6K6W) have been deposited in the Protein Data Bank.
