# Supplementary material for: Structural and biochemical characteristics of two Staphylococcus epidermidis RNase J paralogs RNase J1 and RNase J2
Source: J Biol Chem. 2021 Jan 13;295(49):16863–76. doi: 10.1074/jbc.RA120.014876 (PMC7864078; doi:10.1074/jbc.RA120.014876)
Supplement: Supplementary file 1 [file mmc1.pdf]

Supplementary Material

Tables

Table S1. Comparison of the active site cavity in characterized RNase J enzymes.

PDB ID	Organism	Protein	Ligand	Res (Å)	Cavity Volume ($Å^3$)
3ZQ4 (3)	*Bacillus subtilis*	RNase J1	Zn^{2+}, Ca^{2+}	3.0	2649.2
5A0T (4)	*Streptomyces coelicolor*	RNase J	PEG, Zn^{2+}	2.28	2326.9
3T3N (5)	*Thermus thermophilus*	RNase J	RNA, Zn^{2+}	3.09	1826.4
3T3O (5)		RNase J	RNA, Gly, Zn^{2+}	2.5	1944.8
3BK1 (6)		RNase J	SO_4, Gly, Zn^{2+}	2.33	1446.2
3BK2 (6)		RNase J	UMP, SO_4, Gly, Zn^{2+}	2.1	1393.4
4XWT (7)	*Deinococcus radiodurans*	RNase J	UMP, Gly, Zn^{2+}, Mn^{2+}	2.0	1934.4
4XWW (7)		RNase J	RNA, Gly, Zn^{2+}, Mn^{2+}	1.7	2013.0
5HAA (8)	*Methanolobus psychrophilus*	RNase J	SO_4, Zn^{2+}	2.9	2148.8
5HAB (8)		RNase J	SO_4, (RNA)	2.3	2452.6
	Staphylococcus epidermidis	RNase J2	Mn^{2+}	2.7	2096.0
		RNase J1	Mn^{2+}	3.2	3108.6

Table S2. List of expression constructs

	Tag	Construct	Vector	Primers (5' to 3')
1	C-His_6	*rnj1*	pET 22b	Fw Pr: ATATATGCTAGCATGAAACAACTACATTCAAATGAAGTAGGT GTATA
				Rv Pr: ATATATCTCGAGTTACTTATCTTCATTTACTTTCATTATCACAGGTAAAA
2	N-His_6	*rnj1*	pET Duet-1/MCSI	Fw Pr: ATATATGGATCCGATGAAACAACTACATTCAAATGAAGTAGGTGTATA
				Rv Pr:

				ATATATAAGCTTTTAAATATCACCTATACCACTGCCATCAAC
3	N-His$_6$	*rnj1ΔCTD*	pET Duet-1/MCSI	Fw Pr: ATATATGGATCCGATGAAACAACTACATTCAAATGAAGTAGGTGTATA
				Rv Pr: ATATATAAGCTTTTAAATATCACCTATACCACTGCCATCAAC
4	N-His$_6$	*rnj2*	pET Duet-1/MCSI	Fw Pr: ATATATGGATCCGATGAGTTTAATAAAGAAAAAAAATAAAGATATTCGTATT
				Rv Pr: ATATATAAGCTTTTAGATCTCCGATATGACTGGAATAATCAT
5	None	*rnj1*	pET Duet-1/MCS2	Fw Pr: GGCCAATTGATGAAACAACTACATTCAAATG<br>Rv Pr: GCCTCGAGTTACTTATCTTCATTTACTTTCATTATCAC

Table S3. Primers for site directed mutagenesis

Mutant	Vector	Primers (5' to 3')
RNase J1 D78A	pET Duet-1/MCSI	Fw Pr: ATTACTCATGGACACGAAGCCCATATAGGTGGTGTG
		Rv Pr: CACACCACCTATATGGGC TTCGTGTCCATGAGTAAT
RNase J1 H79A	pET Duet-1/MCSI	Fw Pr: ATTACTCATGGACACGAA GAC GCT ATAGGTGGTGTGCCCTTC
		Rv Pr: GAAGGGCACACCACCTAT AGC GTC TTCGTGTCCATGAGTAAT
RNase J1 D78A|H79A	pET Duet-1/MCSI	Fw Pr: ATTACTCATGGACACGAAGCCGCTATAGGTGGTGTGCCCTTC
		Rv Pr: GAAGGGCACACCACCTATAGCGGCTTCGTGTCCATGAGTAAT
RNase J1 H364A	pET Duet-1/MCSI	Fw Pr AGTAAAATTTCAAACATTGCCACTTCTGGACACGGT
		Rv Pr: ACCGTGTCCAGAAGT GGC AATGTTTGAAATTTTACT
RNase J1 S366A	pET Duet-1/MCSI	Fw Pr: AACATTCACACT GCT GGACACGGTTCTCAA
		Rv Pr: TTGAGAACCGTGTCC AGC AGTGTGAATGTT
RNase J1 H368A	pET Duet-1/MCS	Fw Pr ATTCACACTTCTGGA GCC GGTTCTCAAGGTGAT
		Rv Pr: ATCACCTTGAGAACC GGC TCCAGAAGTGTGAAT

	I	
RNase J2 H80A	pET Duet-1/MCS I	Fw Pr: ACACACGGTCATGAA GCT GCTATAGGCGCAGTA
		Rv Pr: TACTGCGCCTATAGC AGC TTCATGACCGTGTGT
RNase J2 E166A	pET Duet-1/MCS I	Fw Pr: GGTTCTATAGTTTATACTGGA GCG TTTAAGTTTGATCAA
		Rv Pr: TTGATCAAACTTAAA CGC TCCAGTATAAACTATAGAACC
RNase J2 H80A|E166 A	pET Duet-1/MCS I	The H80A background was used to make H80A and E166A double mutant
RNase J2 S368A	pET Duet-1/MCS I	Fw Pr: AAGAAAATTCATGCGGCAAGTCATGGTTGTATGGAAGAA
		Rv Pr: TTCTTCCATACAACC ATG ACT TGC CGCATGAATTTTCTT
RNase J2 S369A	pET Duet-1/MCS I	Fw Pr: AAGAAAATTCATGCG TCA GCT CAT GGTTGTATGGAAGAA
		Rv Pr: TTCTTCCATACAACC ATG AGC TGA CGCATGAATTTTCTT
RNase J2 H370A	pET Duet-1/MCS I	Fw Pr: AAGAAAATTCATGCG TCA AGT GCT GGTTGTATGGAAGAA
		Rv Pr: TTCTTCCATACAACC AGC ACT TGA CGCATGAATTTTCTT

Table S4. Primers for qRT-PCR

**S. N.**	**Organism**	**gene**	**Primers (5' to 3')**
1	*Staphylococcus epidermidis*	*rnj1*	Fw Pr: GATATATCAAAGCGCCACCAG
			Rv Pr: GATAATGCAGCCATA GGTTCAC
2		*rnj2*	Fw Pr: CATGCTATAGGCGCAGT
			Rv Pr: GGGCCTTCATTGCTTCT
3	*Staphylococcus aureus*	*rnj1*	Fw Pr: GCACCACCTGAAACATTTAT
			Rv Pr: TACCTGGGATAGGTGATGAA
4		*rnj2*	Fw Pr: GCGCAACATAAGCATAAAAT
			Rv Pr: TTTAATTCTTCCATGCAACC
5		*16s rRNA*	Fw Pr: TGTCGTGAGATGTTGGG
			Rv Pr: CGATTCCAGCTTCATGT

Figures

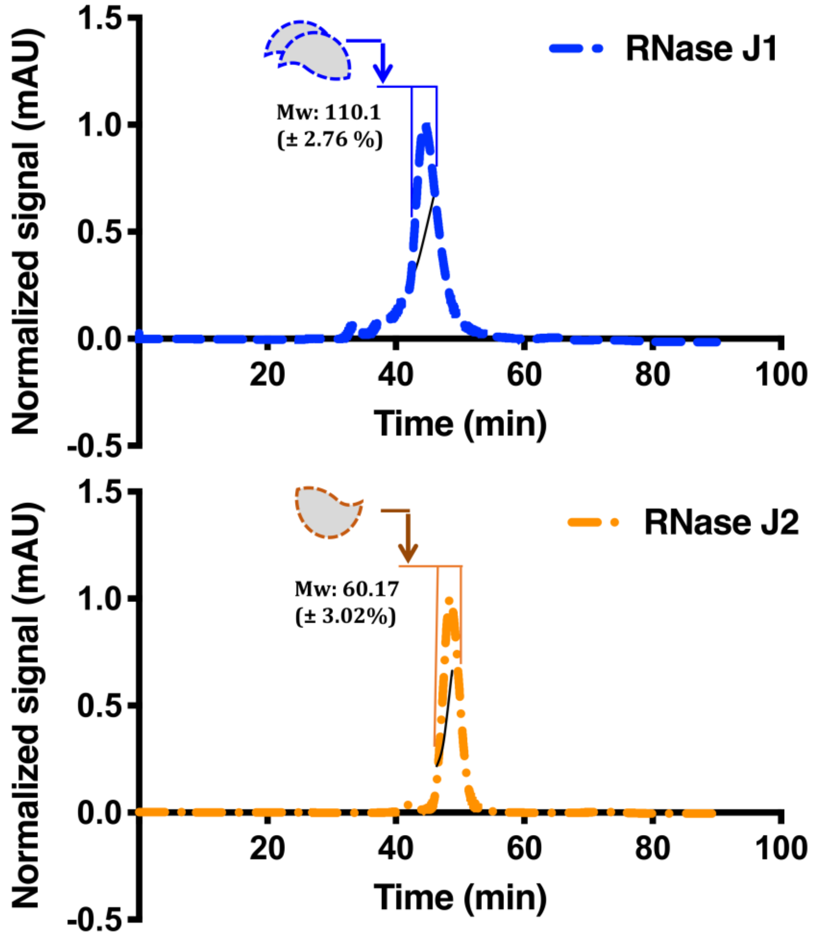

RNase J1		RNase J2	
**Molar Mass Moments (G/mol)**		**Molar Mass Moments (G/mol)**	
Mn	9.465X10^4 (±1.959%)	Mn	5.319X10^4 (±1.902%)
Mp	6.363X10^4 (±1.107%)	Mp	4.073X10^4 (±0.857%)
Mv	n/a	Mv	n/a
Mw	1.101X10^5 (±2.769%)	Mw	6.017X10^4 (±3.020%)
Mz	1.274X10^5 (±6.919%)	Mz	6.869X10^4 (±7.950%)
**Polydispersity**		**Polydispersity**	
Mw/Mn	1.163 (±3.392%)	Mw/Mn	1.131 (±3.569%)
Mz/Mn	1.347 (±7.191%)	Mz/Mn	1.296 (±8.175%)

Figure S1. Size exclusion Chromatography-Multi-Angle Light Scattering (SEC-MALS) of RNase J1 and RNase J2.

Size exclusion chromatography (SEC) on freshly purified enzyme samples was performed using a Superdex 200 column (GE Healthcare). This experiment was performed on a miniDAWN instrument (Wyatt Technologies, Inc).

Legends: M_n (Number average molecular weight): M_n is the statistical average molecular weight of all the polymer chains in a sample. $M_n = \frac{\sum N_i M_i}{\sum N_i}$ where *Mi* = Molecular weight of a subunit (polymer) and *Ni* = Number of chains of that subunit (polymer)

M_w (Weight average molecular weight): Mw considers the molecular weight of a chain in determining contributions to the molecular weight average. $M_w = \frac{\sum N_i M_i^2}{\sum N_i M_i}$

M_z (Z-average molecular weight): Mz is measured in sedimentation equilibrium experiments. Z stands for centrifugation. It is also known as higher average molecular weight.

$M_w = \frac{\sum N_i M_i^{n+1}}{\sum N_i M_i^n}$ (where n=2)

M_P (Molecular weight of highest peak): Mp is used for narrowly distributed (monodisperse) proteins such as standards used in calibration of a column.

M_V (Velocity average molar mass): Used for gaseous states and invokes kinetic energy to determine molecular weight.

Polydispersity index: Used as a measure of broadness of a molecular weight distribution of a polymer (represented by Mw/Mn). For a monodisperse polymer, Mw/Mn should be equal to 1.0.

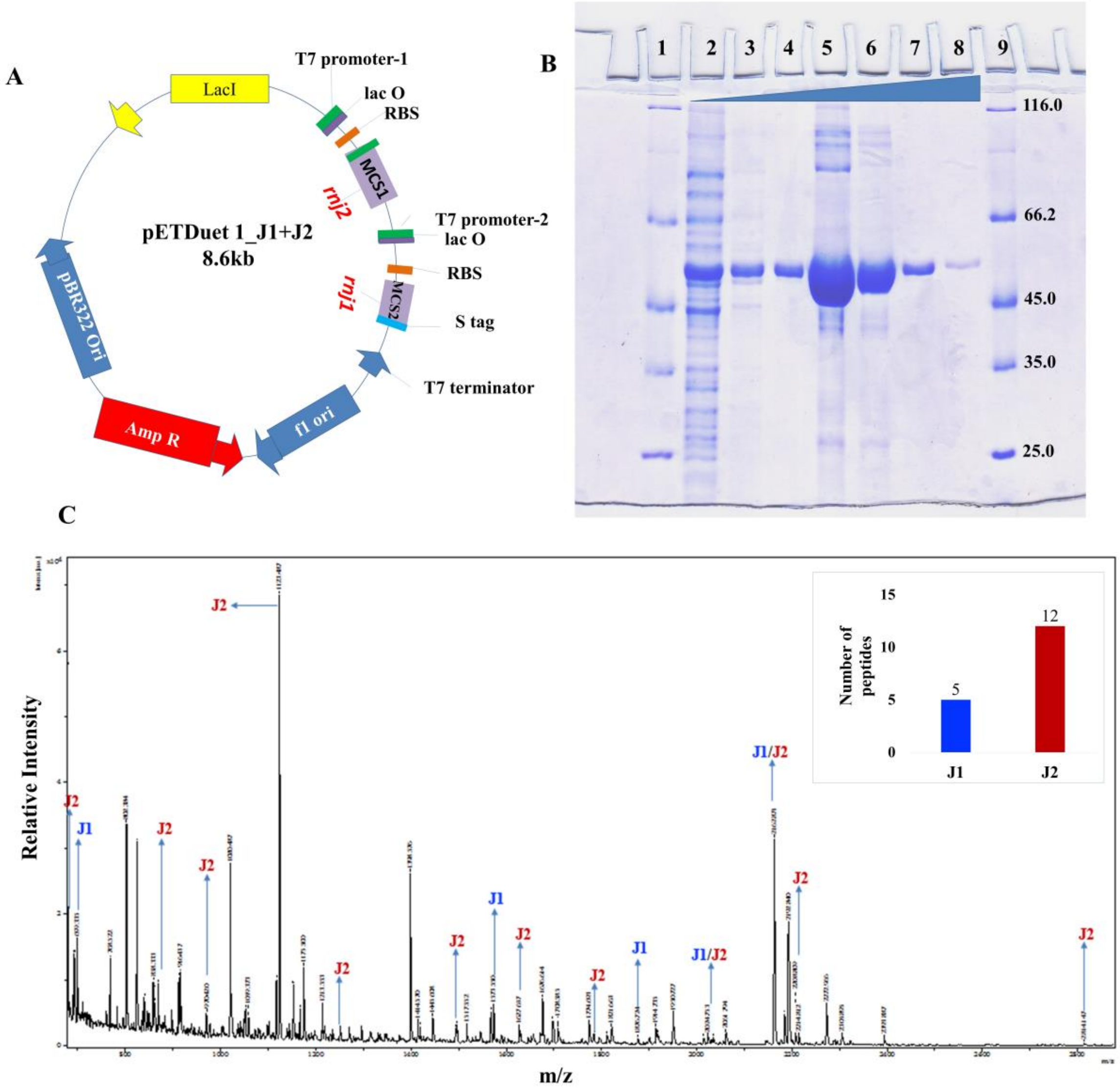

MALDI-MS based peptide mapping of co-purified RNase J1 and RNase J2

Protein Name	Fragment	m/z (Obs.)	MH+ (Calc.)	Peptide sequence
	116-129	1573.5	1573.6	TAKLNEINEDSVIK
	252-257	693.3	693.3	IVTFGR
RNase J1	380-394	1876.7	1876.7	IIKPKYFLPIHGEYR
	490-505	2034.7	2034.8	GFVYMRESGQLIYDAQR
	490-507	2162.8	2162.9	GFVYMRESGQLIYDAQRKI
	116-133	2034.8	2034.7	DKKVRYYTVNNDSIMRFK
	169-182	1627.6	1627.7	FDQSLHGHYAPDEK
	241-243	1496.7	1496.7	IQQVLNIASKLNR
	255-260	678.3	678.3	VSFLGR
RNase J2	255-270	1783.7	1783.7	VSFLGRSLESSFNIAR
	261-270	1123.4	1123.5	SLESSFNIAR
	261-271	1251.5	1251.6	SLESSFNIARK
	272-279	970.4	970.4	MGYFDIPK
	437-456	2208.8	2208.8	VNSGNILIDGIGIGDVGNIV
	498-504	862.3	862.4	ESEDLLR
	522-538	2162.8	2162.9	RIEWSEIKQNMRDQISK
	534-557	2814.1	2814.1	DQISKLLFESTKRPMIIPVISEI

Figure S2. **A.** Schematic of the vector map of the construct co-expressing *rnj1* and *rnj2* in the pETDuet 1 vector (Novagen, Inc). **B.** The co-expressed proteins were co-purified by immobilized metal affinity chromatography. RNase J2 has a poly-histidine tag at the N-terminus. In this protein preparation, the lysis buffer contained 50mM Tris HCl (pH 8.0),150mM NaCl, 2mM β-ME and 10% glycerol. The protein bound to the Ni-NTA affinity resin was eluted by a gradient of imidazole (5-250mM). Lane 1, 9 is the molecular weight marker; Lane 2: Flow through; Lanes 3, 4: protein in the wash fractions (two column volumes of the lysis buffer containing 5mM Imidazole); Lanes 5-8 protein fractions that were eluted with increasing concentration of imidazole. **C**. The eluted protein was digested with 20μg/ml trypsin (Sigma-Aldrich, Inc) and the fragments were analyzed by MALDI-TOF mass spectrometry (Bruker Daltonics, Inc). While fragments corresponding to both RNase J1 (blue) and RNase J2 (brown) could be identified, the lower concentration of RNase J1 in the eluted fraction precluded further use of this protein preparation for biophysical studies. The bar graph (inset) provides a graphic representation of the relative levels of the two paralogues in the co-purified protein preparation. The peptides that could be unambiguously mapped on to the sequence of *S. epidermidis* RNase J1 and RNase J2 are listed. The stoichiometric excess of RNase J2 in this sample suggests that RNase J2 can exist as a monomer on its own.

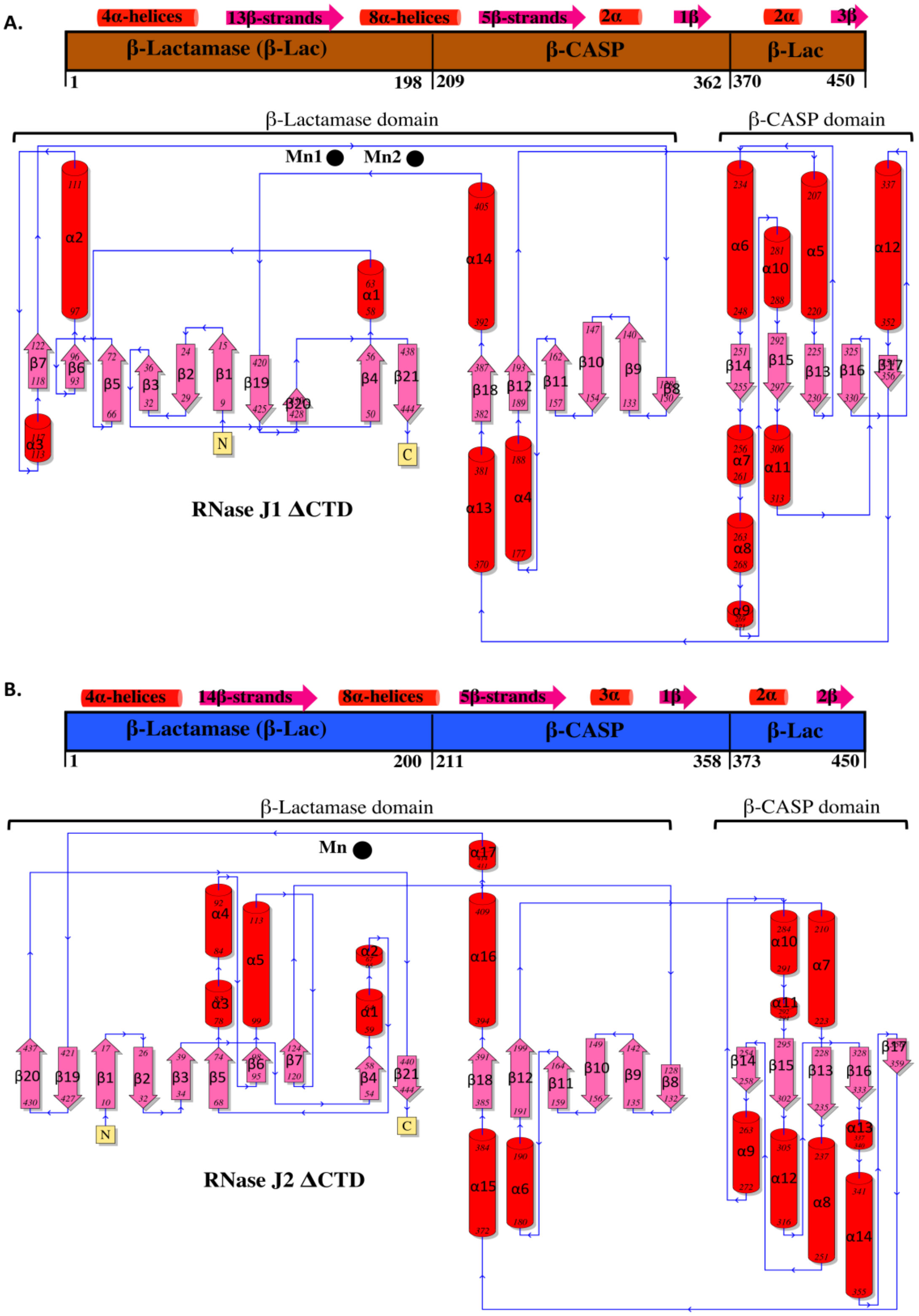

Figure S3. Domain architecture and topology of **A**. RNase J1 and **B**. RNase J2. The topology diagram was prepared using the PDBsum-web server (2).

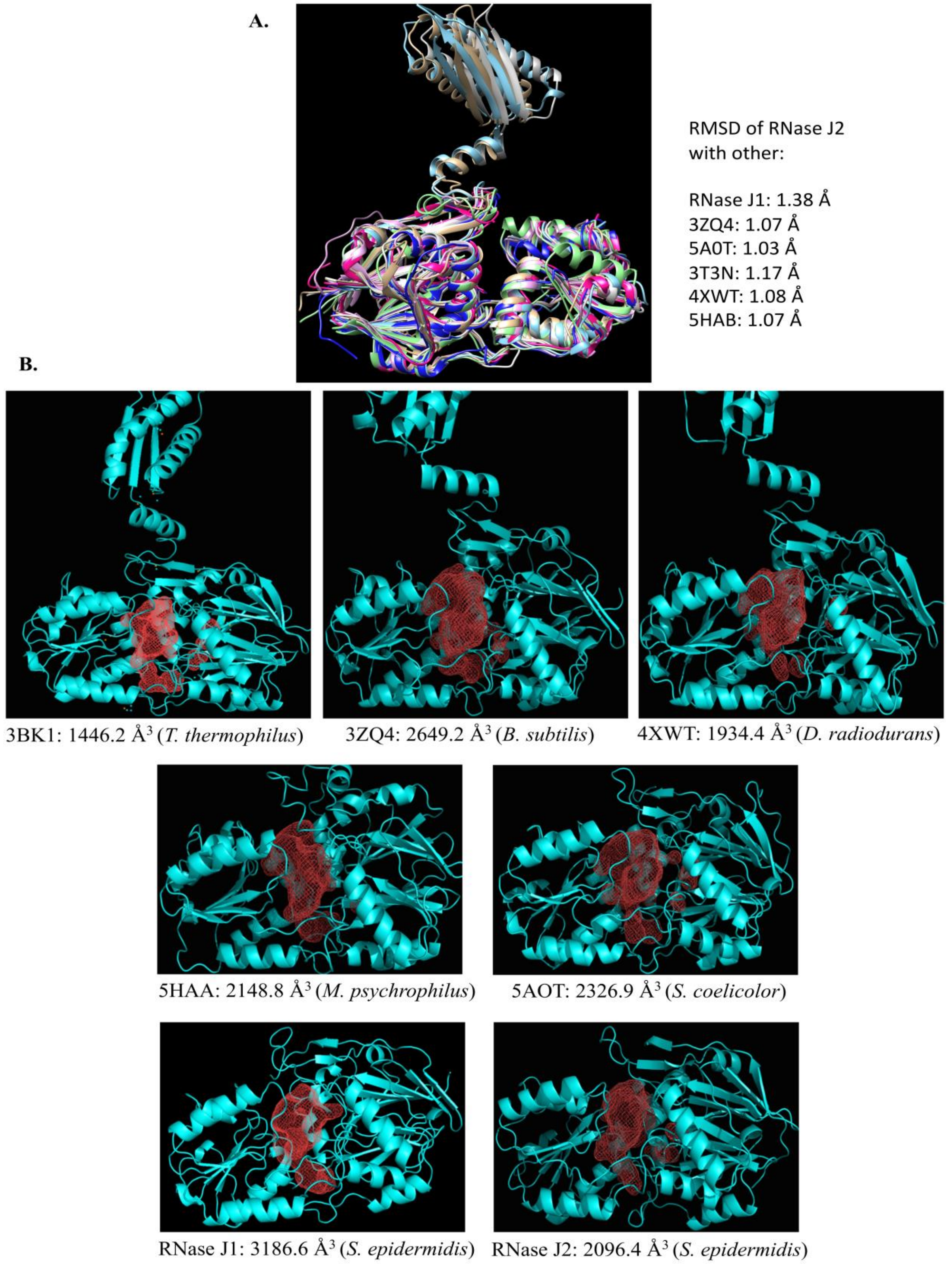

Figure S4. Representation of the active site pocket in RNase J1, RNase J2 and characterized RNase J homologues. **A.** Superposition of RNase J2 (blue) with RNase J1 (grey), 3ZQ4 (pink), 5A0T (green), 3T3N (brown), 4XWT (tan) and 5HAB (magenta). The C_α average root mean square deviation is *ca* 1.1. **B.** The active site cavity (red mesh in each structure)

was generated using HOLLOW (3). Cavity volumes are compiled in Supplementary Table S1.

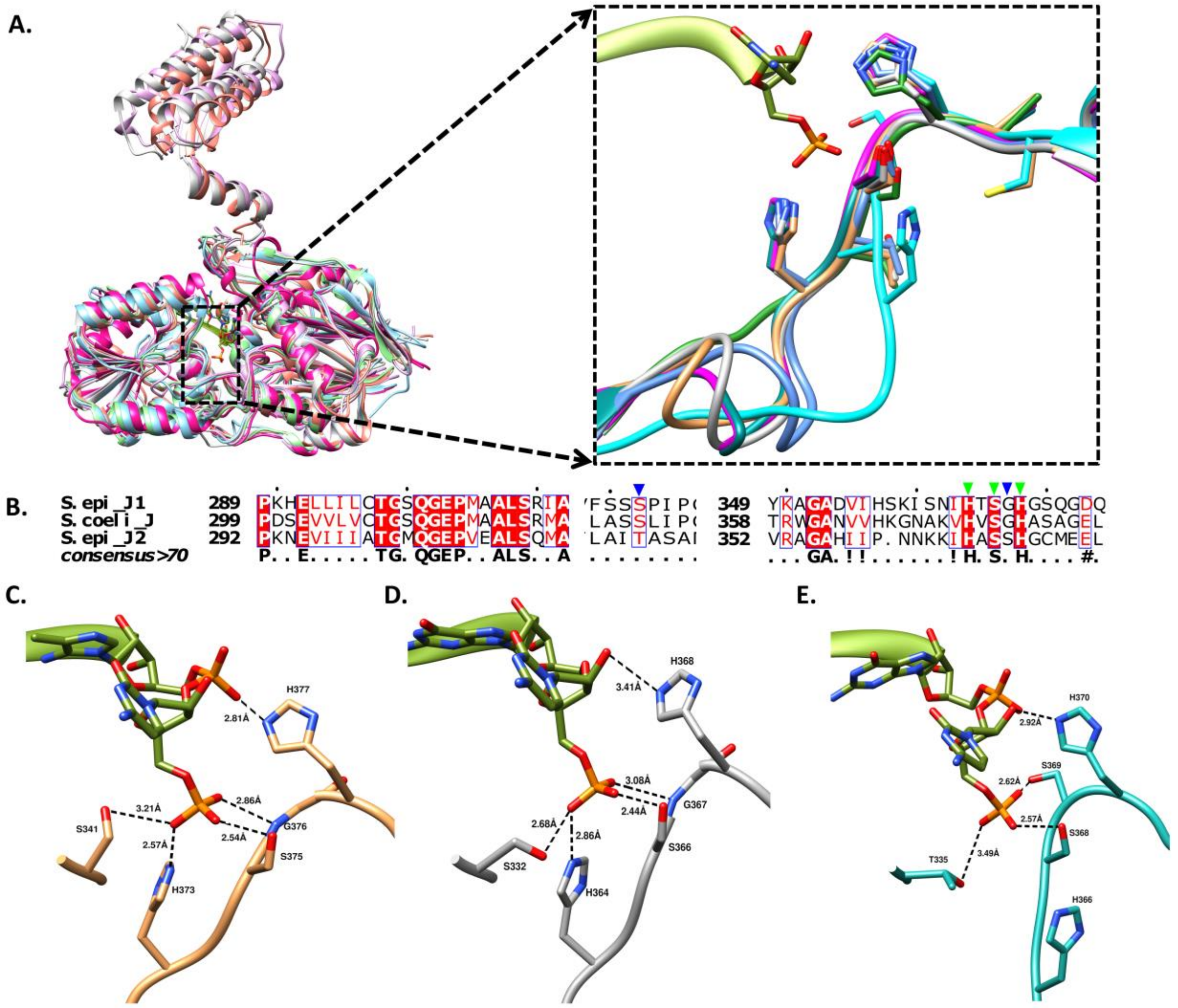

Figure S5. 5' end sensing is mediated by a loop that connects the β-CASP to the β-lactamase domain. **A.** Superimposed structures of RNase J2 (cyan) with RNase J1 (grey) and five different PDBs: 3ZQ4 (pink), 5A0T (green), 3T3N (brown), 4XWT (tan) and 5HAB (magenta). **B.** Sequence alignment of RNase J2/ RNase J1 with *S. coelicolor* RNase J (5A0T) shows that residues that interact with 5' monophosphate are mostly conserved. In this sequence alignment, green triangles depict conserved residues whereas blue triangles highlight substitutions in RNase J2. **C.** The monophosphate sensing region in *S. coelicolor* RNase J. **D.** Residues involved in 5' monophosphate recognition in RNase J1. **E.** Residues involved in 5' monophosphate recognition in *S. epidermidis* RNase J2. The sequence alignment was performed in ESPript 3.0 (4) while the structural representations were made using UCSF Chimera (5).

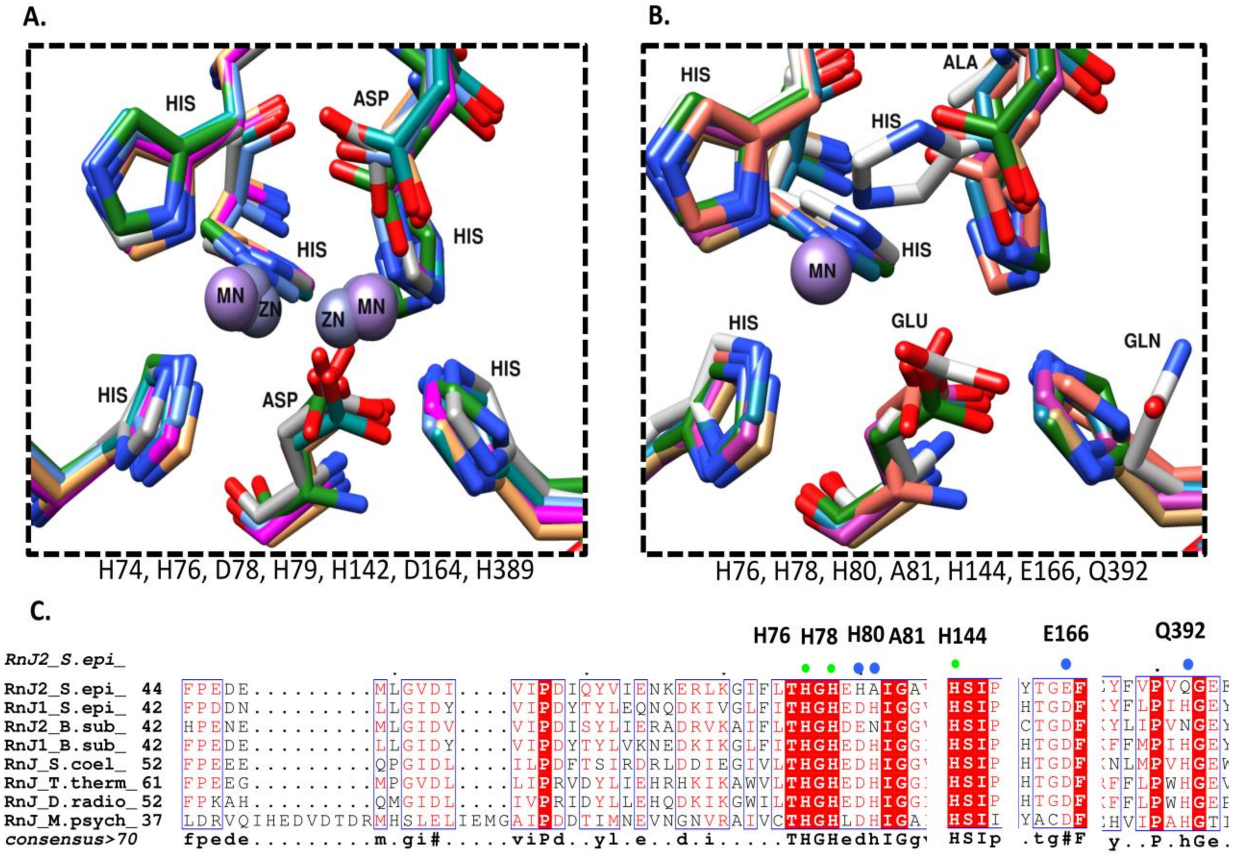

Figure S6. A. Superposition of active site of RNase J1 (grey) with five RNase J homologues. The relevant residues from RNase J1 are noted below the structural superposition. **B.** Superposition of active site of RNase J2 (grey) with five RNase J homologues. Residues at the active site of RNase J2 are noted. **C.** Sequence alignment of RNase J2 with RNase J1 and other homologs. In this alignment, green dots represent conserved residues while blue dots show substitutions in RNase J2. This sequence alignment was prepared using ESPript 3.0 (4) and the molecular representations were made using UCSF Chimera (5)

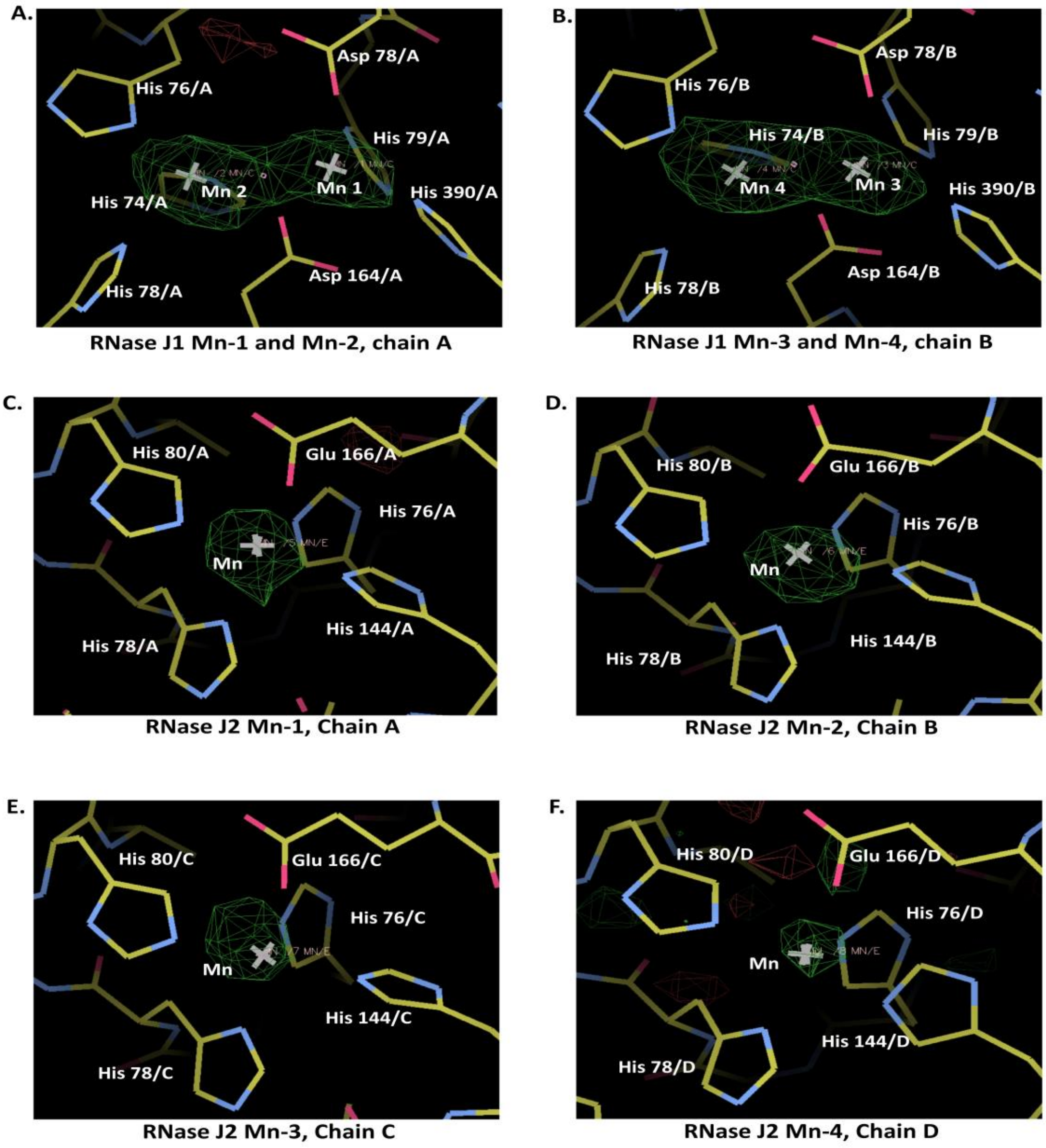

Figure S7. Anomalous difference Fourier electron density maps show the bound metal ion (Mn^{2+}) at the active site of RNase J1 and RNase J2. A and B. In the case of RNase J1, the data was collected at a synchrotron source. **C-F**. The diffraction data for this crystal was collected at a home source (Rigaku FRE X-Ray generator and Raxis IV detector) at 1.54 Å. There are four molecules of RNase J2 in the asymmetric unit of the crystal. Snapshots of the electron density maps are shown for all four active sites.

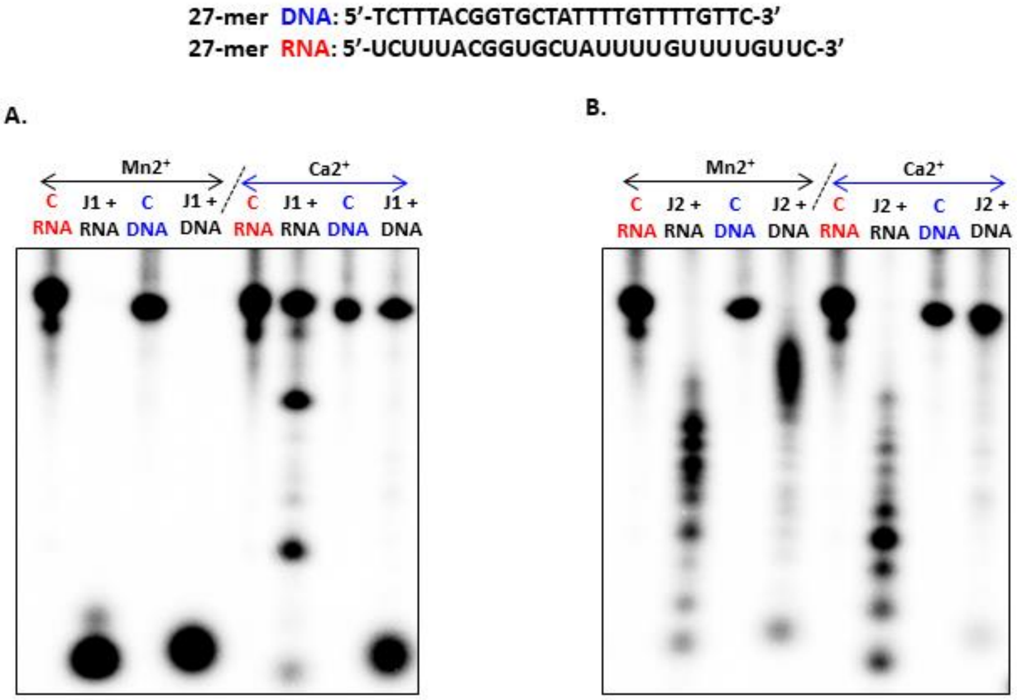

Figure S8. The *Staphylococcal* RNase J paralogues show DNase activity

A comparison between the DNase and RNase activity of RNase J1 and RNase J2. The sequences of 27-mer RNA and 27-mer DNA substrates is shown. The RNA substrate used in this experiment is identical to that used in the characterization of the *S. aureus* RNase J1 (1). **A.** Both RNase and DNase activity of RNase J1 prefer Mn^{2+} as a metal co-factor**.** We note trace endo-nuclease activity on the RNA substrate and poor exonuclease activity on the DNA substrate in the presence of Ca^{2+}. **B.** In the case of RNase J2, while the RNase activity is relatively higher in the presence of Ca^{2+} (when compared to Mn^{2+}), DNase activity is higher in the presence of Mn^{2+}. As stated in the manuscript, RNase J2 is a less active enzyme when compared to RNase J1. The concentration of RNase J2 is thus higher in these assays (72µM) when compared to RNase J1 (600nM).

20-mer-RNA: 5'-ACUGGACAAAUACUCCGAGG-3'

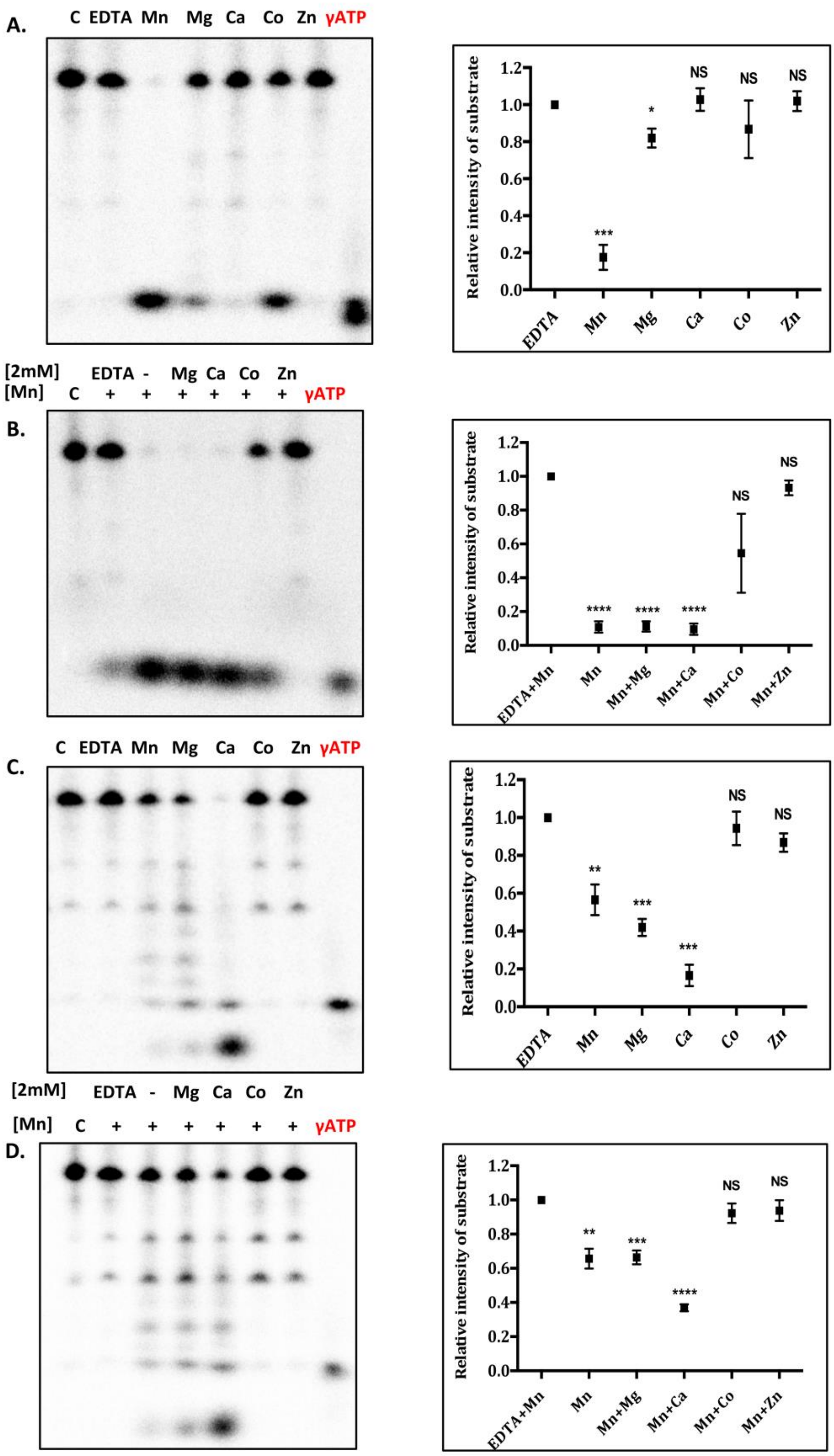

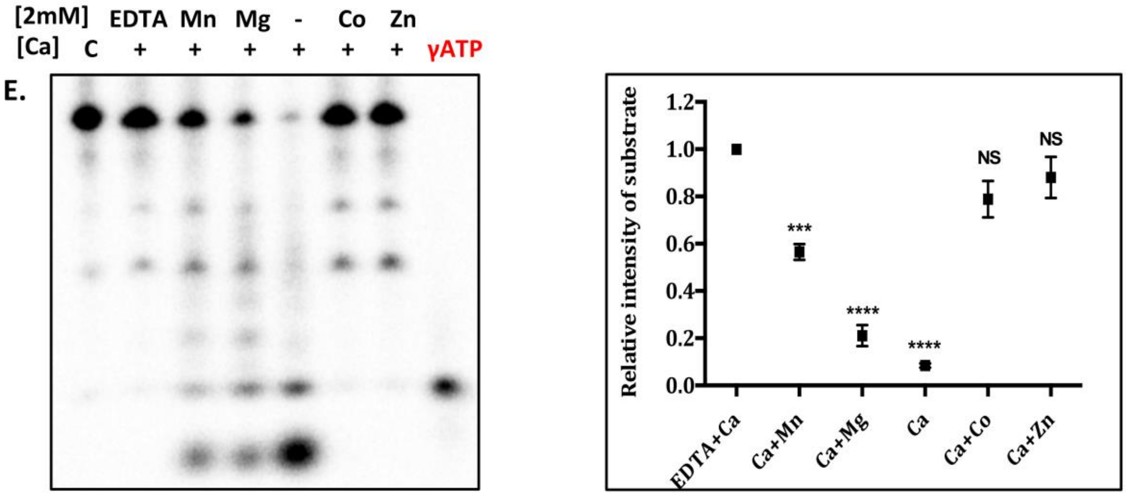

Figure S9. Influence of metal cofactors on the RNase activity of RNase J1 and RNase J2. A. Activity of RNase J1 in the presence of different metal ions. The sequence of the 20-mer RNA substrate used in this assay is shown. The first lane is the control (no enzyme) while the second is the reaction mixture with a chelator (EDTA). The subsequent lanes show reactions performed in the presence of different metal ions. Decrease in substrate concentration was highest in the presence of Mn^{2+}. Statistics from an unpaired *t*-test are noted (shown as mean ± s.e.m). These are- **P* < 0.05, ***P* < 0.01, ****P* < 0.001, *****P* < 0.0001, NS: not significant. **B.** A control activity assay where Mn^{2+} is constant (1 mM; depicted by "+" on each lane) while the other metal ions are maintained at 2 mM concentration**.** The quantification of the data from the 20 % Urea PAGE is shown in the adjacent panel. Zn^{2+} inhibits catalytic activity of RNase J1. Unpaired *t*-test with different competing metal ions in presence of Mn^{2+}, data shown as mean ± s.e.m. **C.** Activity assay of RNase J2 in the presence of different metal cofactors. The first lane is the control (without enzyme) while the second is with the chelator, EDTA. Subsequent lanes show reactions performed in the presence of different metal ions. **D.** A competition assay for comparison between different metal co-factors for RNase J2 activity. While addition of Ca^{2+}, Mg^{2+} and Mn^{2+} showed significant decrease in the intensity of the band corresponding to the substrate, Co^{2+} and Zn^{2+} does not seem to influence catalytic activity. **E.** A control activity assay where Ca^{2+} is constant (1 mM; shown by the "+" sign on the top of each lane) while the other metal ions are maintained at 2 mM concentration**.** The data is shown as mean ± s.e.m.

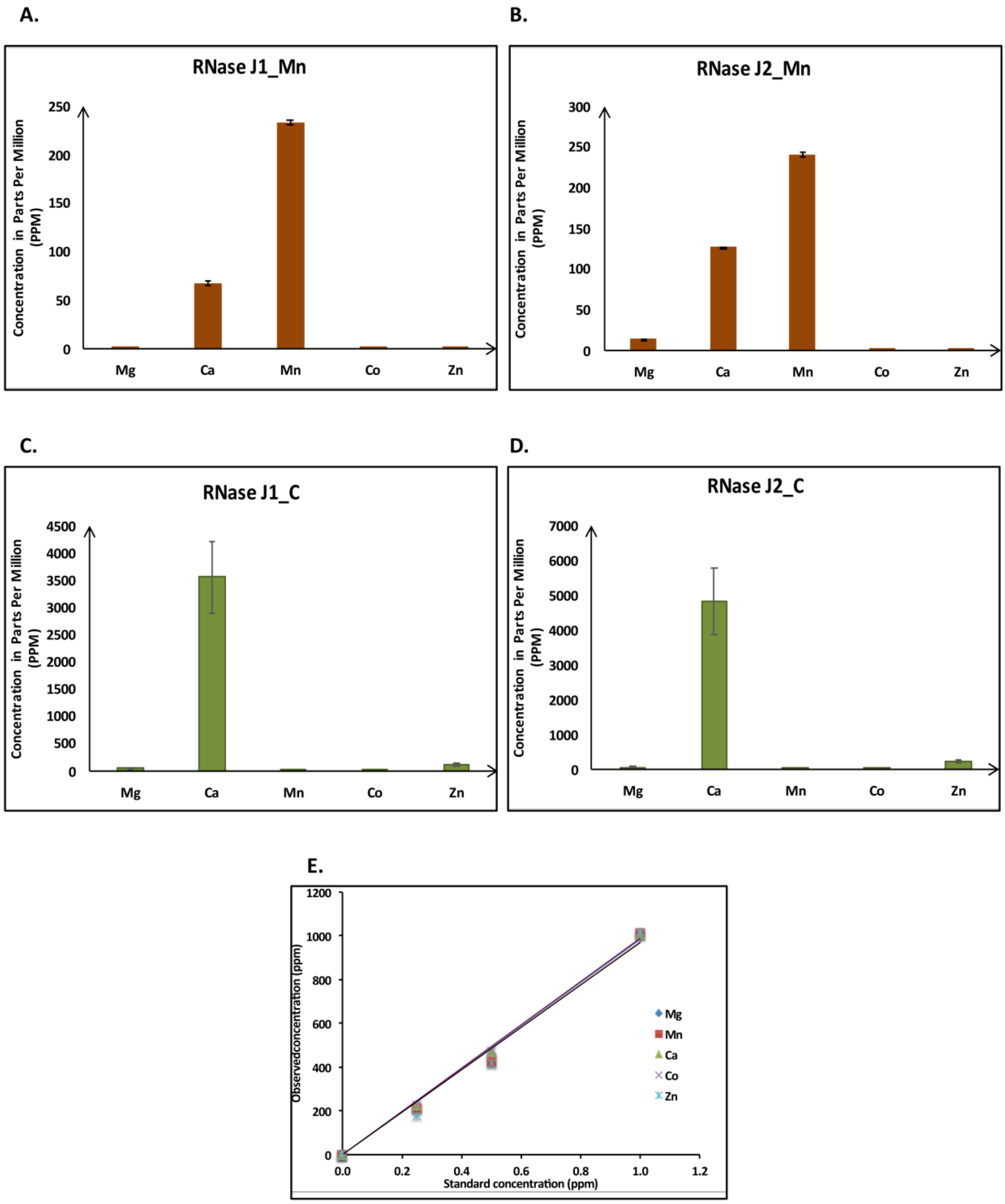

Figure S10. Inductively coupled plasma mass spectrometry (ICP-MS) analysis of protein samples. **A**. Freshly purified RNase J1 samples show the presence of bound Mn^{2+}, Ca^{2+} and a trace Mg^{2+}, Co^{2+} and Zn^{2+}. **B.** Freshly purified RNase J2 samples show the presence of bound Mn^{2+}, Ca^{2+} and a trace Mg^{2+}, Co^{2+} and Zn^{2+}. **C**. Replacement of metal ions for activity assays evaluated by ICP-MS. Chelation of Mn^{2+} and subsequent replacement by Ca^{2+} in the case of RNase J1 (**C**) and RNase J2 (**D**). This information was essential to evaluate enzyme samples used in activity assays and validate the experimental protocol for metal ion replacement prior to biochemical analysis.

Strains	Source	SCC*mec* type	PVL	Agr	ST/CC	Genome ID	Reference(s)
LVP-2	Corneal scrapping	V	+	II	772 (CC1)	AOFV00000000	PMID:22291460 PMID:24722327
LVP-7	Orbital abscess	V	+	III	88 (CC88)	Not sequenced	PMID:22291460
GH-3989	Pneumonitis	V	+	II	772 (CC1)	ALWH00000000	PMID:22548694 PMID:24722327
COL	HA-MRSA	I	-	I	250 (CC8)	CP000046	PMID:15774886
ATCC 12228	ATCC strain	SSC*pbp4*	-	I	2 (CC2)	CP022247	PMID:28883148
AEH-333	Corneal ulcer	V	+	II	772(CC1)	ALWF00000000	PMID:22291460
VH-60	Nasal carrier	V	+	II	772(CC1)	ALWG00000000	PMID:22548694 PMID:24722327
LVP-1	Corneal scrapping	MSSA	-	III	1(CC1)	Not sequenced	PMID:22291460

*SCC*mec*- Staphylococcal cassette chromosome *mec*, PVL-Paton-Valentine Leucocidin,

Agr- Accessory gene regulator.

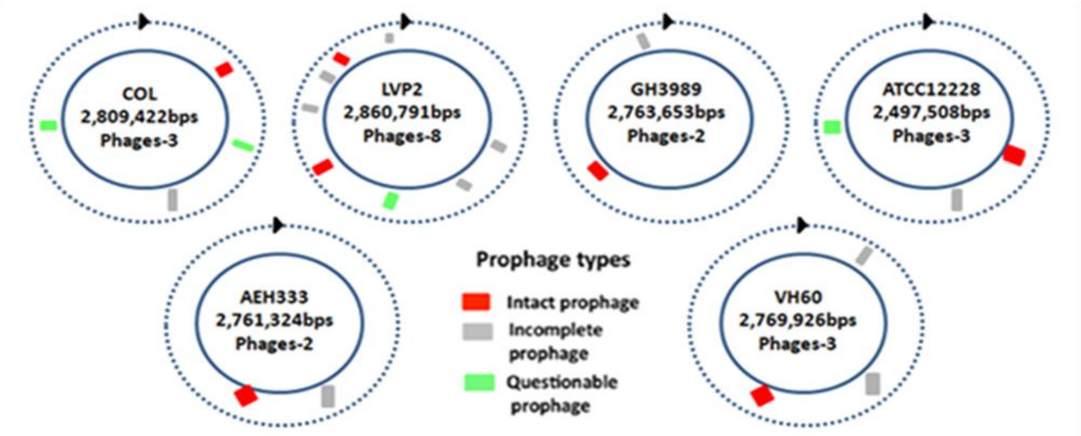

Figure S11. Compilation of the clinical, genotypic and prophage signatures in the different *Staphylococcal* strains examined in this study. The prophage signatures in the clinical isolates were predicted using the genome sequences (6,7,8). The phage search tool (PHAST) was used for this sequence search (9).

References

1. Hausmann, S., Guimaraes, V.A., Garcin, D., Baumann, N., Linder, P. and Redder, P. (2017) Both exo- and endo-nucleolytic activities of RNase J1 from *Staphylococcus aureus* are manganese dependent and active on triphosphorylated 5'-ends. *RNA Biol*, **14**, 1431-1443.

2 Laskowski, R.A., Jablonska, J., Pravda, L., Varekova, R.S. and Thornton, J.M. (2018) PDBsum: Structural summaries of PDB entries. *Protein Sci*, **27**, 129-134.

3 Ho, B.K. and Gruswitz, F. (2008) HOLLOW: generating accurate representations of channel and interior surfaces in molecular structures. *BMC Struct Biol*, **8**, 49.
4. Robert, X. and Gouet, P. (2014) Deciphering key features in protein structures with the new ENDscript server. *Nucleic Acids Res*, **42**, W320-324.
5. Pettersen, E.F., Goddard, T.D., Huang, C.C., Couch, G.S., Greenblatt, D.M., Meng, E.C. and Ferrin, T.E. (2004) UCSF Chimera--a visualization system for exploratory research and analysis. *J Comput Chem*, **25**, 1605-1612.
6. Nadig, S., Velusamy, N., Lalitha, P., Kar, S., Sharma, S. and Arakere, G. (2012) *Staphylococcus aureus* eye infections in two Indian hospitals: emergence of ST772 as a major clone. *Clin Ophthalmol*, **6**, 165-173.
7. Balakuntla, J., Prabhakara, S. and Arakere, G. (2014) Novel rearrangements in the *staphylococcal* cassette chromosome mec type V elements of Indian ST772 and ST672 methicillin resistant Staphylococcus aureus strains. *PLoS One*, **9**, e94293.
8. Shambat, S., Nadig, S., Prabhakara, S., Bes, M., Etienne, J. and Arakere, G. (2012) Clonal complexes and virulence factors of *Staphylococcus aureus* from several cities in India. *BMC Microbiol*, **12**, 64.
9. Zhou, Y., Liang, Y., Lynch, K.H., Dennis, J.J. and Wishart, D.S. (2011) PHAST: a fast phage search tool. *Nucleic Acids Res*, **39**, W347-352.
